# Breeding Novel Chemistry in Willow: New Hetero Diels–Alder Cyclodimers from Arbusculoidin and Salicortin Suggest Parallel Biosynthetic Pathways

**DOI:** 10.3390/plants13121609

**Published:** 2024-06-11

**Authors:** Clarice Noleto-Dias, Charlotte Lomax, Alice Bellisai, Gianluca Ruvo, Claudia Harflett, William J. Macalpine, Steven J. Hanley, Michael H. Beale, Jane L. Ward

**Affiliations:** 1 Plant Sciences & the Bioeconomy, Rothamsted Research, West Common, Harpenden AL5 2JQ, UK; 2 Protecting Crops and the Environment, Rothamsted Research, West Common, Harpenden AL5 2JQ, UK; william.macalpine@rothamsted.ac.uk

**Keywords:** arbusculoidin, isoarbusculoidin, miyabeacin, *Salix* spp., salicinoid, biosynthesis

## Abstract

An investigation of phenolic glycosides extracted from *Salix* germplasm revealed that arbusculoidin (benzyl 1-*O*-β-d-glucopyranosyl-1-hydroxy-6-oxo-2-cyclohexenyl carboxylate) and its enolic 6-glycoside isomer, isoarbusculoidin, are widespread across the Salix family. An analysis of natural hybrid species and progeny from a willow breeding programme demonstrated that the putative biosynthetic pathway leading to the salicinoid family of phenolic glycosides runs in parallel to a “benzyl”-based pathway to arbusculoidin. The introduction of a known Diels–Alder reaction trait from *Salix dasyclados*, as well as an acylation trait, into progeny containing both salicyl- and benzyl- pathways caused the formation of all possible hetero-cyclodimers from mixtures of reactive dienone (acyl)glycosides that participated in cross-over reactions. In addition to providing access to new analogues of the anti-cancer dimer miyabeacin, the analysis of the breeding progeny also indicated that these dienone (acyl)glycosides are stable in planta. Although the immediate biosynthetic precursors of these compounds remain to be defined, the results suggest that the (acyl)glycosylation reactions may occur later in the pathway than previously suggested by in vitro work on cloned UGT enzymes.

## 1. Introduction

**Figure 1:**
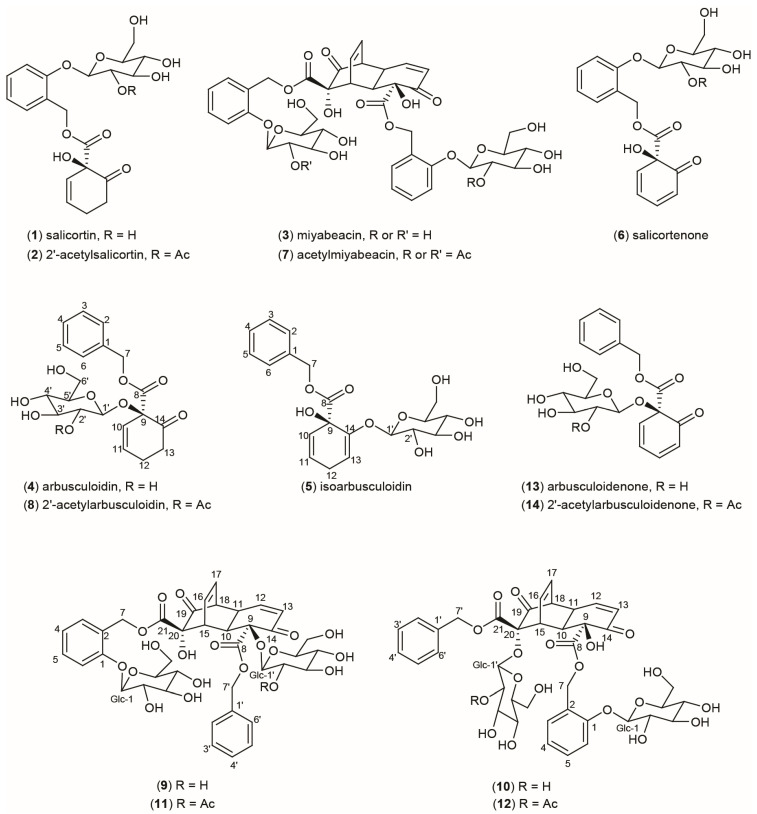
Compound structures. Structure numbering system does not reflect biosynthetic provenance; for clarity in NMR assignments, numbering was maintained for each structural module.

**Figure 2:**
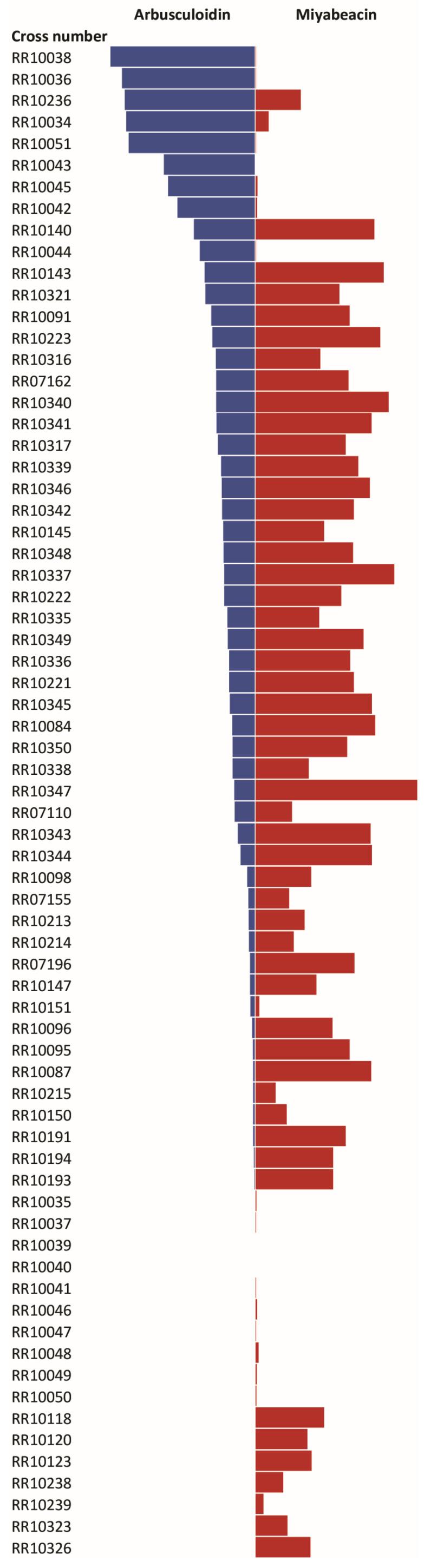
Comparison of miyabeacin and arbusculoidin levels observed in LC-MS analysis of polar (80:20 water:methanol) extracts of stem tissue from breeding progeny of trial ‘RR/CS/722’. Bars represent integrated peak areas corresponding to miyabeacin (**3**) and arbusculoidin (**4**) in negative-mode MS following reversed-phase HPLC.

The metabolome of the Salicaceae family of trees and shrubs (*Salix*, *Populus*, and *Chosenia*) is characterised by a range of abundant phenolic glucosides that includes the salicinoids, a subgroup of pharmacological importance [1,2,3]. Structurally, this class of metabolites is based on salicin (salicyl alcohol-2-glucoside) and more complex examples, such as salicortin (**1**) (Figure 1) and 2′-acetylsalicortin (**2**). Salicortin and the acylated derivatives are major extractable metabolites in many species of willow and poplar, and these compounds have become of biosynthetic interest as they contain the unusual 1-hydroxy-6-oxo-2-cycohexenyl 1-carboxylate (HCH) ring structural fragment. It is this reactive moiety that forms the basis of the newly discovered [3] anti-cancer dimeric salicinoid miyabeacin (**3**). The HCH group is not confined to salicinoids, and it has been known for some time that an analogue of salicortin based on benzyl alcohol exists in some willow species. Evans et al. [4] were the first to report on the structure and occurrence of benzyl 1-*O*-β-d-glucopyranosyl-1-hydroxy-6-oxo-2-cyclohexenyl carboxylate (**4**) and the isomer (**5**), which bears an enolic glucopyranosyl function, in *Salix arbusculoides* (Alaskan little tree willow). We are now naming these metabolites arbusculoidin (**4**) and isoarbusculoidin (**5**). These two compounds have become of interest to us in light of recent biochemical and genetic investigations into the proposed biosynthetic network leading to salicortin [5], in which the involvement of acyltransferases and the intermediacy of benzylbenzoate and/or salicyl-7-benzoate has been implicated [6]. Furthermore, the early involvement of a glycosyltransferase (UGT71L1/2/3), that glycosylates salicyl-7-benzoate in vitro has been suggested in both willow and poplar [7,8]. Gene knockouts in transgenic poplar have confirmed this UGT enzyme to be a key player in the in vivo pathway to salicortin [7,8]. Consideration of the benzyl alcohol-based structures of arbusculoidin and isoarbusculoidin, (**4**) and (**5**), however, suggests that there may be a parallel pathway from benzylbenzoate whereby formation of the HCH ring must precede glycosylation, which, in these molecules, occurs on the HCH moiety.

Further pieces of the biosynthetic jigsaw have emerged from the discovery of miyabeacin (**3**) from *Salix miyabeana* and *S. dasyclados* [3]. Miyabeacin has a diketo-1,4-ethenodecalin core structure and is the product of an intermolecular [4+2] Diels–Alder reaction between two units of an ephemeral dienone monomer called “salicortenone” (**6**) that is closely related to salicortin. In our previous report [3], it was also demonstrated that the biosynthetic process required to produce (**6**) and, subsequently, this new family of dimeric molecules in *Salix* spp. is a heritable trait that is genetically encoded in *S. miyabeana* and *S. dasyclados* and their progeny. In vivo cross-over [4+2] reactions between acylated analogues of (**6**) in hybrid willows generated derivatives such as acetyl miyabeacin (**7**), indicating that both glycosylation and acylation preceded the Diels–Alder reaction to miyabeacin. 

In this paper, we turn our attention to the apparent parallel biosynthetic pathway leading to arbusculoidin (**4**) and isoarbusculoidin (**5**). We describe the quantitation of these compounds across a *Salix* species diversity panel and report the chemical structures of acetylarbusculoidin (**8**) and four new [4+2] cyclodimers of the miyabeacin family (**9**–**12**) formed in hybrid willows via condensation of dienone analogues of salicortin (**1**) and arbusculoidin (**4**). We define the genetic sources of three metabolic traits—(i) presence of the arbusculoidin pathway, (ii) acetylation of the glucose, and (iii) the formation of the dienone precursor and subsequent spontaneous [4+2] dimerisation reactions, and show how these metabolic traits can be merged by hybridisation to produce novel structures in *Salix*. Cross-over Diels–Alder products show that pools of reactive glycosylated dienones from both salicortin and arbusculoidin pathways must co-exist in planta. However, the exact biosynthetic relationship (precursor versus product) between the dienones salicortenone (**6**) and arbusculoidenone (**13**) and the corresponding saturated ketones salicortin (**1**) and arbusculoidin (**4**) still remains to be accurately defined. 

## 2. Results

### 2.1. Salicortin Analogues Based on Benzyl Rather than Salicyl Rings Are More Common than Expected

Authentic samples of arbusculoidin (**4**) and isoarbusculoidin (**5**) were isolated by preparative HPLC from a living accession (NWC1165) of *Salix arbusculoides* held in the UK National Willow Collection (NWC). Structures were characterised by ^1^H-NMR and high-resolution LC-MS, and are in agreement with those given in the literature [4]. To assess the natural ranges and levels of these analogues relative to salicortin in *Salix* genotypes across the NWC, we mined the ^1^H-NMR fingerprinting data of aqueous methanolic extracts of stem tissue from 191 willow accessions harvested at the dormant stage (February) [9]. Data from 7 accessions, harvested when plants had senesced (November), were also included in the study for comparison. The results of quantitation via NMR integration of compound-specific resonances against internal standard are shown in Table 1. Arbusculoidin (**4**) was detected in 35 accessions at levels varying from 35.35 ± 1.76 to 3.05 ± 0.08 mg/g dry weight in stems, while isoarbusculoidin (**5**) was quantified in only six of the accessions in concentrations ranging from 33.25 ± 3.05 to 3.83 ± 0.30 mg/g d.w. (Table 1). There was no obvious correlation between the levels of these metabolites and the levels of salicortin (**1**), which itself varied between undetectable and 111.27 ± 51.49 mg/g d.w. There was also no proportional relationship between the amounts of arbusculoidin (**4**) and isoarbusculoidin (**5**) produced. Accessions which produced high amounts of (**4**) at levels well in excess of salicortin (**1**) included NWC617 and NWC614 (both *S. schwerinii*) and the established commercial biomass variety “Resolution” (NWC1124), which also contains some *S. schwerinii* in its pedigree. None of these accessions accumulated (**5**), which was observed in its highest quantity in dormant stems of *S. saposhnikovii* (NWC1239). In this accession, isoarbusculoidin was also predominant over arbusculoidin. In NWC1165, an accession of *S. arbusculoides*, similar levels of (**4**) and (**5**) were produced, which was in agreement with data reported for this species [4]. Interestingly, several genotypes produced isoarbusculoidin only, and these accessions included NWC1060 *S. gmelinii*; NWC1270 *S. triandra*; and, at low concentrations, NWC577 *S. dasyclados.* Of these, NWC1270 *S. triandra* was the only accession to produce isoarbusculoidin (**5**) in the absence of salicortin (**1**). Inspection of the LC-MS data of the polar extracts of *S. schwerinii* ‘K3 Hilliers’ (NWC615) and *S. saposhnikovii* (NWC1239) (Supplementary Figure S1) indicated that arbusculoidin (**4**) and isoarbusculoidin (**5**) eluted at 24.32 min and 21.88 min, respectively. Both compounds exhibited strong [M + formate − H]^−^ ions at *m*/*z* 453.1389 corresponding to the molecular formula C_21_H_25_O_11_. In the case of arbusculoidin (**4**), a clear [M − H]^−^ ion at *m*/*z* 407.1333 (C_20_H_23_O_9_) was also observed. Further analysis of the total ion chromatogram of NWC615 indicated a peak at 29.11 min with an [M + formate − H]^−^ ion at *m*/*z* 495.1501, corresponding to a new compound with molecular formula C_22_H_26_O_10_, 42 mass units higher, which is characteristic of an acetylated derivative. The isolation of this metabolite and a comparison of its ^1^H-NMR data to (**4**) are shown in Table 2. Key differences were the presence of peaks corresponding to an acetyl moiety accompanied by a downfield shift of the double doublet at δ 4.77 (J = 8.0, 9.5) compared to the corresponding signal (δ 3.41) in (**4**). Interestingly, the ^13^C chemical shift of C-2′ was not significantly affected, although this is in line with previous studies on acetylated salicin analogues [9] where ^13^C signal differences were only observed when acetylation occurred at the C-6′ position and not at the C-2′ position. These data, together with key HMBC correlations, indicated that the acetyl moiety in (**8**) was attached at C-2′ hydroxy group of the glucose. 

The position of acetylation was consistent with other salicinoids observed in *Salicaceae*. Whilst other derivatives can also show acetylation at the C-6 position of the glucose, this is often as a result of acyl migration following extraction or treatment in basic conditions [10,11]. The absolute configuration of the glucose in (**4**) was previously determined by Evans et al. [4], after cation exchange resin acid hydrolysis, to be the D enantiomer, and the assignment of the β-anomer followed from the H-1′/H-2′ coupling constant (7.5 Hz). The absolute configuration at C-9 in (**4**) and (**8**) was assumed to be (S) following from Feistel et al. [12], who determined this via circular dichroism for a number of HCH-bearing salicinoids, including salicortin (**1**).

### 2.2. Novel Dimers Arising from Arbusculoidin Producing Salix Genotypes Hybridising with ‘Diels–Alder’ Miyabeacin-Producing Genotypes

**Figure 3:**
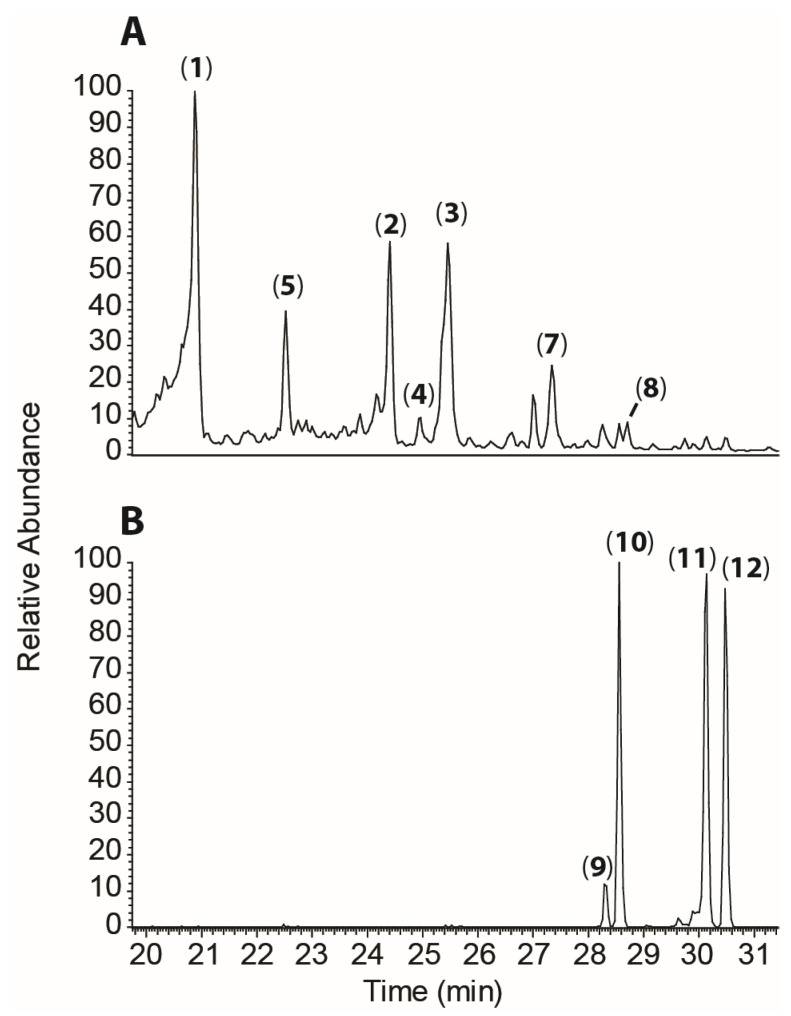
LC-MS analysis of RR10143 in negative ion mode. (**A**) TIC (19.4–31.4 min). (**1**): salicortin; (**2**): 2′-acetylsalicortin; (**3**): miyabeacin; (**4**): arbusculoidin; (**5**): isoarbusculoidin; (**7**) acetyl miyabeacin; (**8**): 2′-acetylarbusculoidin. (**B**) Extracted ion chromatograms for novel dimers, using mass range *m*/*z* 827–870. Numbers refer to structures in Figure 1.

Our previous work [3] has already identified [4+2] dimeric structures, e.g., miyabeacin (**3**), in *Salix miyabeana* and *S. dasyclados*, as well as that alternate acyl-glucose derivatives (acetylated, benzoylated) of these dimers can be formed by conventional crossing via careful parental selection. To assess the effect of combining the benzyl (versus salicyl)-containing series, exemplified by hybridisation of arbusculoidin (**4**)-producing genotypes with accessions known to produce miyabeacin family dimers, we assessed the pedigrees of genotypes bred at Rothamsted Research as part of the BEGIN biomass improvement programme [13]. Within the available progeny from trial ‘RR/CS/722’, 70 examples contained either *S. dasyclados* or *S. miyabeana* in their pedigree, and thus were potentially capable of producing miyabeacin (**3**) (Supplementary Table S1). The panel was screened using LC-MS in negative ion mode to determine the levels of arbusculoidin (**4**) and, thus, the potential to produce novel dimeric entities via Diels–Alder cycloaddition in planta (Figure 2). The highest contents of arbusculoidin (**4**) were found in breeding progeny RR10038 and RR10036, both arising from a cross between RR08083 (NWC607 *S. rehderiana* × NWC619 ‘Lapin’) and NWC446 *S. aegyptiaca*. However, despite containing high levels of salicinoids, these hybrids did not produce any miyabeacin (**3**) or other dimeric compounds, suggesting that the Diels–Alder capability was not present (or not operating) in these progeny. When the miyabeacin (**3**) content was also considered alongside the arbusculoidin content, two hybrids (RR10140 and RR10143) contained significant peaks for both markers. Both hybrids represented siblings from a cross between ‘RR08083’ (NWC607 *S. rehderiana* × NWC619 ‘Lapin’) and NWC577 *S. dasyclados* ‘77056’. Inspection of the LC-MS data from RR10143 displayed four additional, higher-molecular-weight peaks in the total ion chromatogram (Figure 3), as well as peaks for salicortin (**1**), 2′-acetyl salicortin (**2**), arbusculoidin (**4**), isoarbusculoidin (**5**), acetyl arbusculoidin (**8**), miyabeacin (**3**), and acetyl miyabeacin (**7**). For chemical structure determination, a larger-scale extraction of RR10143 was performed, and semi-preparative HPLC enabled isolation of each novel entity. Structures were elucidated using MSMS and NMR.

**Figure 4:**
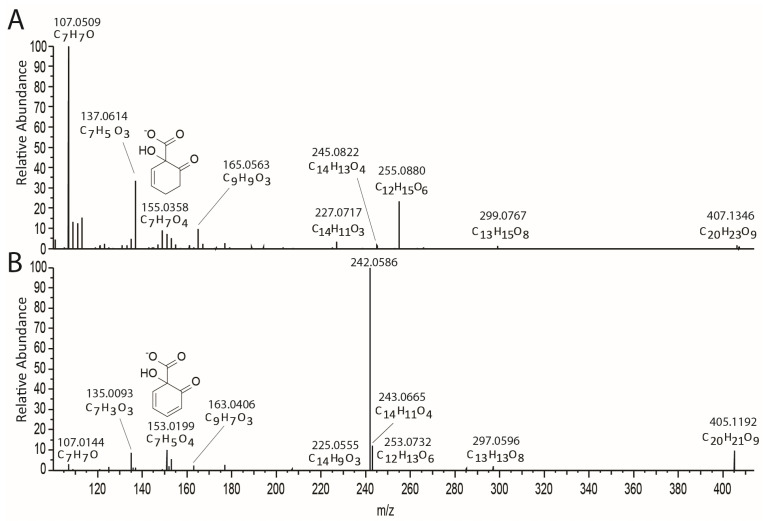
Comparison of the MSMS fragmentation arising from (**A**) arbusculoidin (**4**) *m*/*z* 407 and (**B**) the *m*/*z* 405 fragment of (**10**).

In the total ion chromatogram from LC-MS, compounds **9** and **10** eluted at 28.2 and 28.5 min, respectively, and each showed an [M − H]^−^ ion at *m*/*z* 827.2390, corresponding to compounds with a molecular formula of C_40_H_44_O_19_. Two peaks appeared later at 30.0 (**11**) and 30.4 min (**12**), and each showed an [M − H]^−^ ion at *m*/*z* 869.2499, corresponding to derivatives with molecular formula C_42_H_46_O_20_. MSMS spectra of these four entities generated a suite of common fragments appearing at *m*/*z* 109 (C_6_H_5_O_2_), 123 (C_7_H_7_O_2_), 237 (C_8_H_13_O_8_), 277 (C_18_H_13_O_3_), 317 (C_20_H_13_O_4_), 321 (C_19_H_13_O_5_), 355 (C_20_H_15_O_5_), and 515 (C_26_H_27_O_11_), suggesting that they represented a panel of structurally related molecules. In our previous structural determination of the dimeric compound miyabeacin (**3**) [3], the MSMS analysis of a smaller monomer ion (*m*/*z* 421, salicorteneone (**6**)) resulting from in-source fragmentation of the parent (MW 844) confirmed the salicinoid nature of the monomeric units (Supplementary Figure S2). The corresponding monomer ion (*m*/*z* 463) relating to 2′-acetyl-salicortenone was also present in the LC-MS data of acetyl and diacetyl miyabeacin analogues (Supplementary Figures S2 and S3). A similar approach was taken with (**9**) and (**10**) (Supplementary Figure S4). Here, in addition to the observed [M − H]^−^ at *m*/*z* 827.2390, we observed a monomer-related ion at *m*/*z* 421.0752, corresponding to (**6**), and a second at *m*/*z* 405.1192. A comparison (Figure 4) of the MSMS of this ion to that of the [M − H]^−^ ion of arbusculoidin (**4**) revealed a series of fragments that were two mass units lower across the series (*m*/*z* 297 vs. 299; 253 vs. 255; 163 vs. 165; 153 vs. 155; 135 vs. 137) and suggested that these structures arise from a Diels–Alder reaction of salicortenone (**6**) and arbusculoidenone (**13**), the dehydro analogue of arbusculoidin (**4**).

^1^H and ^13^C spectra of **9**–**12** were compared to published NMR data [3] and this suggested that **9**–**12** all possessed the same diketo-1,4-ethenodecalin core, identical to the [4+2] Diels–Alder “core” structure present in the cyclodimer miyabeacin (**3**). Consistent ^1^H signals for the enone hydrogen atoms H-12 and H-13 were observed at δ 6.63–6.44 and 6.08–6.03, respectively. Signals for the olefinic hydrogens H-16 and H-17 were also present at δ 6.23–6.00 and 5.94–5.91, respectively. In all ^1^H spectra, signals for 4 methine hydrogens, H-10, H-11, H-15, and H-18, were observed with δ values between 3.90 to 3.41 ppm (Table 3). The main differences between the data of (**9**–**12**) and (**3**) pointed towards a variation in the position and nature of the glucose attachment.

**Figure 5:**
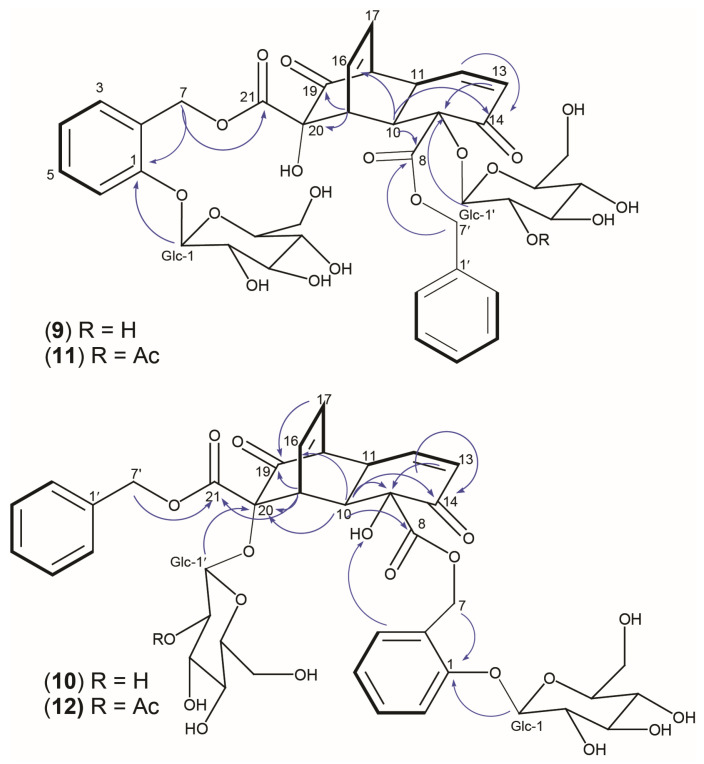
^1^H-^1^H COSY (bold line) and selected ^1^H-^13^C HMBC correlations (H → C) (blue arrows) for **9** to **12**.

To elucidate the relative configuration and exact structures, NMR data were examined more closely. In the case of data for (**9**), the signals relating to the core moiety showed good agreement (chemical shift and coupling constants) with those of miyabeacin (**3**), with the exception of the signal for C-9, which showed a downfield shift of 6.3 ppm (δ_C_ 88.5 ppm). Further connections were established by 2D NMR (COSY, TOCSY, HSQC, and HMBC). A ^1^H-^13^C HMBC correlation from the anomeric proton Glc-1 (δ 4.35) to C-9 indicated that glucose was directly attached to the core and explained the resonance shift for C-9. The position of H-10 (δ 3.87) was elucidated by a TOCSY experiment, where correlations among all protons of this spin system were detected. Both H-7′ (δ 5.30 and 5.22) and H-10 showed HMBC correlations with C-8, confirming their position in relation to the core structure. The benzylic methylene signals were present as H_2_-7′ (δ 5.30 and 5.22), while the other two sets of doublets H_2_-7 (δ 5.37 and 5.15) were representative of the salicyl moiety attached to the core. The specific position of each hydrogen and carbon in the molecule was achieved through COSY and HMBC analysis, and key correlations are highlighted in Figure 5. With this, we concluded that compound **9** had arisen via a [4+2] Diels–Alder cyclodimerisation reaction between salicortenone (**6**), acting as the diene, and arbusculoidenone (**13**), behaving as a dienophile.

Compound (**10**) is an isomer of (**9**), and although their NMR data are very similar, we confirmed using 2D NMR techniques that they differ in regiochemistry. In **10**, we observed a 5.5 ppm downfield shift of the ^13^C signal of C-20 (δ_C_ 85.5 ppm), indicating a C-20-glucose attachment in this derivative. This was further corroborated by the presence of an HMBC correlation between the anomeric proton Glc-1′ (δ 4.71) and C-20. The methylene protons H_2_-7 (δ 5.41 and 5.18) showed HMBC correlations with C-1 (δ_C_ 158.2) and C-8 (δ_C_ 173.8), confirming that the salicyl moiety was attached to C-8. COSY and key HMBC correlations for (**10**) are also shown in Figure 5. Compound (**10**) is therefore suggested to be biosynthesised via a cyclodimerisation reaction between arbusculoidenone (**13**), acting as the diene, and salicortenone (**6**), behaving as a dienophile.

The LC-MS data of (**11**) and (**12**) were suggestive of acetylated derivatives of (**9**) and (**10**). Inspection of their NMR data (Table 3) confirmed that compound (**11**) was the Glc-2′-acetyl derivative of (**9**). New resonances at δ_H/C_ 2.12 (s)/23.1 and δ_C_ 175.8 arose from the methyl and carbonyl portions of the acetyl moiety. Placement of the acetate group was elucidated by HMBC experiments and a correlation of the acetyl carbonyl to a double doublet at δ 4.76 (J = 8.0, 9.4), assigned to Glc-2′. The glucose directly attached to the dimer core structure. This indicates that (**11**) represented a hybrid dimer formed via the Diels–Alder cycloaddition of salicortenone (**6**) and 2′-acetylarbusculoidenone (**14**). This was further confirmed via the inspection of the LC-MS spectra for (**11**), which showed an in-source fragment at *m*/*z* 447, (Supplementary Figure S5) corresponding to 2′-acetylarbusculoidenone (C_22_H_23_O_10_) and 42 mass units higher than the unacetylated arbusculoidenone monomer normally appearing at *m*/*z* 405. Compound (**12**) was the Glc-2′-acetyl of (**10**). The new acetate resonances were observed at δ_H/C_ 2.12 (s)/23.1 and δ_C_ 175.8, relating to the methyl and carbonyl portions, respectively. Again, a correlation of the carbonyl signal with the Glc-2′ proton (double doublet at δ 4.76 (J = 8.0, 9.4) placed the acetate on the glucose directly attached to the dimer core structure rather than on the glucose of the salicyl portion in the other half of the molecule. LC-MS again showed the expected in-source fragment at *m*/*z* 447, corresponding to the acetyl arbusculoidenone monomer (Supplementary Figure S5).

The above compounds represented the novel metabolites that could be isolated and structurally characterised by NMR. To investigate whether further (lower abundance) dimeric entities had been formed, we examined the total ion chromatogram and relevant extracted ion spectra of the RR10143 extract. Two further peaks with parent ions at *m*/*z* 869 appeared at 29.62 and 29.78 min. Inspection of their MSMS and associated monomer ions (*m*/*z* 405 and 463) suggested a pair of isomeric compounds generated via the Diels–Alder reaction between acetylsalicortenone and arbusculoidenone (Supplementary Figure S6). Additionally, at 31.76 and 32.04 min, ions with [M − H]^−^ ions at *m*/*z* 911, corresponding to metabolites with formula C_44_H_47_O_21_, were detected. Analysis of the monomer ions (*m*/*z* 463 (not detected) and 447) from in-source fragmentation (Supplementary Figure S7) was indicative of the diacetyl hybrid dimer generated from 2′-acetylsalicortenone and 2′-acetylarbusculoidenone. Also present in the low-abundance peaks was an entity appearing at 23.00 min with [M − H]^−^ at *m*/*z* 811, corresponding to a molecule with molecular formula C_40_H_43_O_18_. Analysis of the in-source fragments revealed a single ion at *m*/*z* 405, corresponding to arbusculoidenone (Supplementary Figure S8). Thus, this compound was putatively assigned as the product of the reaction of two arbusculoidenone monomers, one acting as the dienophile and the other as the diene. Finally (Supplementary Figure S9), traces of two compounds could be detected at 27.41 and 27.53 min. Both entities showed parent ions at *m*/*z* 853, and both showed monomer ions at *m*/*z* 405 and 447, corresponding to arbusculoidenone and 2′-acetylarbusculoidenone.

### 2.3. Parental Composition Provides ‘Substrates’ for Novel Diels–Alder Reaction Products

**Figure 6:**
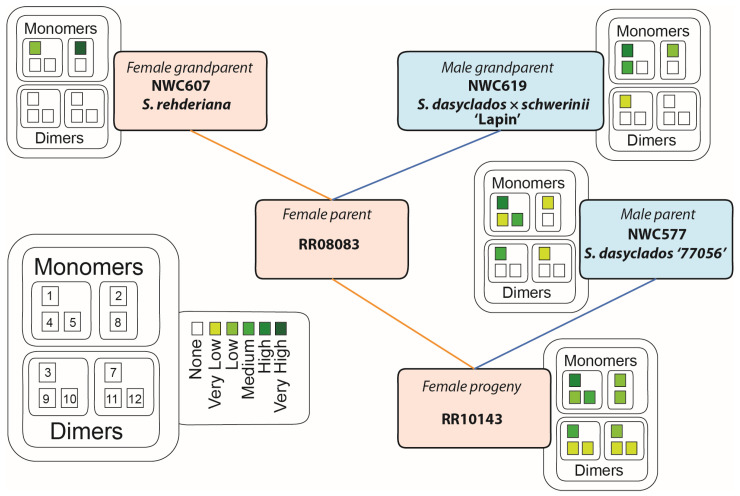
Pedigree of RR10143 indicating male and female parental and grandparental genotypes. Colour-coded levels of observed metabolites are indicated for each available genotype. 1: salicortin (**1**); 2: 2′-acetylsalicortin (**2**); 3: miyabeacin (**3**); 4: arbusculoidin (**4**); 5: isoarbusculoidin (**5**); 7: acetyl miyabeacin (**7**); 8: 2′-acetylarbusculoidin (**8**); 9: (**9**); 10: (**10**); 11: (**11**); 12: (**12**).

The pedigree of RR10143 is (NWC607 *S. rehderiana* × (NWC619 *S. dasyclados* × *schwerinii* ‘Lapin’) ‘RR08083’) × NWC577 *S. dasyclados* ‘77056’ (Figure 6). In order to attribute possible chemical inputs to the final progeny, an LC-MS analysis was conducted of stem tissues of NWC607 (*S. rehderiana*), NWC619 (*S. dasyclados* × *schwerinii* ‘Lapin’), and NWC577 (*S. dasyclados* ‘77056’) (Figure 6 and Supplementary Figure S10), which constituted the male parental and female grandparental material. The LC-MS profile of NWC577 (*S. dasyclados* ‘77056’), the male parent, contained salicortin (**1**) as the major salicinoid, with low levels of 2′-acetylsalicortin (**2**). Miyabeacin (**3**) and low levels of acetylated miyabeacin (**7**) were the only detected dimeric compounds. Other relevant peaks included the presence of arbusculoidin (**4**) and isoarbusculoidin (**5**) in a ratio of 1:20. The germplasm of the female parent, RR08083, was no longer available, but samples of its parents, the grandparents of RR10143, were analysed.

The dominant salicinoid in NWC607 (*S. rehderiana*), the female grandparent, was 2′-acetylsalicortin (**2**), with lower levels of salicortin (**1**). Neither arbusculoidin (**4**) nor isoarbusculoidin (**5**) were present in this accession, and no dimeric compounds were detected. In the case of NWC619 ‘Lapin’, the male grandparent, itself a hybrid of *S. dasyclados* × *schwerinii*, both salicortin (**1**) and 2′-acetylsalicortin (**2**) were present with higher levels of the unacetylated compound (**1**). In addition, the profile of NWC619 ‘Lapin’ also contained significant levels of arbusculoidin (**4**), but not isoarbusculoidin (**5**). In terms of dimeric compounds, low levels of miyabeacin (**3**) and acetylmiyabeacin (**7**) could also be detected. None of the parental/grandparental germplasms produced detectable levels of the new dimer derivatives (**9**–**12**) or 2′-acetylarbusculoidin (**8**). It was, therefore, concluded that the ability to assemble these molecules and the other (putative) minor configurations had arisen as a consequence of the novel mixing of substrates and reactions achieved in the parental hybridisation process. Despite the fact that the female parent of RR10143 was no longer available for analysis, the likely heritage of each structural element in the novel dimers (Figure 6) was traceable through the grandparents and can be summarised as follows: (i) dienone accumulation and resultant Diels–Alder dimerisation capability is derived from *S. dasyclados*; (ii) the expression of the abusculoidin pathway originates from the *S. scherwinii* heritage in the male grandparent, NWC619 ‘Lapin’, from the maternal side; and (iii) the acetylation trait arises from the female grandparent *S. rehderiana*, also on the maternal side.

## 3. Discussion

**Figure 7:**
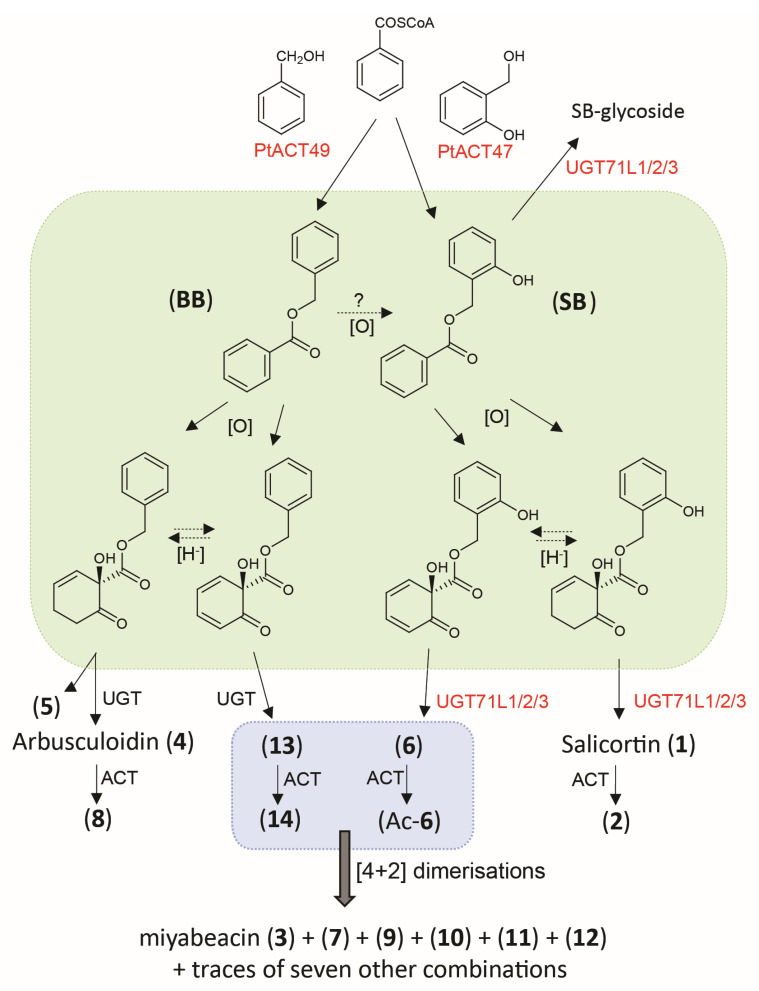
Suggested architecture and parallel relationship of salicyl- and benzyl- based biosynthetic pathways in willow and poplar. Enzymes in red have been previously biochemically characterised in vitro; however, the precise nature and range of their substrates in planta remains to be defined. The green box represents a metabolic channel (metabolon) containing mainly unstable and transient intermediates in an oxidative/reductive chain leading to the HCH ring. Exit from this metabolon is via glycosylation, which, for the salicinoids, has a stabilising effect. The blue box represents a pool of (acyl)glycosyl-dienones that provides the substrates for the observed combinatorial cross-over inter-molecular [4+2] Diels–Alder chemistry. Structure numbers relate to Figure 1.

Our results clearly show that, in many willows, the salicinoid biosynthetic pathway suggested to originate from salicylbenzoate (**SB**) operates in parallel with a homologous pathway based on benzylbenzoate (**BB**). This is in line with the original report from cloned poplar enzymes studied in vitro [6], which stated that two acyltransferases, PtACT49 and PtACT47, for which different substrate preferences have been demonstrated in vitro, may be responsible for the genesis of **BB** and **SB**, the two suggested entry points into the postulated parallel pathways in planta (Figure 7). We cannot, however, rule out the possibility that, in planta, **SB** is formed by hydroxylation of **BB**. Additionally, the two pathways share biosynthetic machinery, i.e., the enzymes required to carry out the transformation of the lower benzoate rings into the HCH function in both salicortin (**1**) and arbusculoidin (**4**). The balance between the two pathways is genotype-dependent in our survey, but likely to be dynamic in expression where relative flux varies due to genetic and/or environmental influences. More detailed time-course experiments will be necessary to investigate these aspects. There was no evidence that the ratio of arbusculoidin to isoarbusculoidin was fixed, and thus, the transformation of the aglycone to the tertiary glycoside in (**4**) or the enol-glycoside in (**5**) may be a result of different UGT enzymes. However, there is also potential that the enol-glycoside is formed initially and then rearranges to the more stable tertiary glycoside. Nevertheless, it is now clear that the glycosylation (and subsequent acetylation of the glucose moiety) occurs after a proposed oxidative dearomatisation of the benzoate ring in benzylbenzoate to give arbusculoidin/isoarbusculoidin (see Figure 7). Extending this logic to the parallel salicortin pathway suggests that the HCH ring may be formed before the glycosylation by the already characterised UGT71L2/3 enzymes, which are known to be involved in the salicinoid pathway [7,8]. These enzymes could act on the upper salicyl ring hydroxy group, leading to the 2-glycosylated dienones, which themselves are the obligate reactants participating in the apparently spontaneous Diels–Alder reaction to miyabeacin (**3**) and its analogues [3]. It remains to be seen whether these particular UGTs have the ability to *ortho*-glycosylate a wider range of salicyl intermediates, such as the aglycone of salicortenone (**6**, aglycone). Our previous experience with the synthesis and handling of **SB** [8] suggests that the formation of the 2-glycoside has a stabilising effect on the ester bond by removal of anchimeric effect of the free 2-hydroxy. We would also suggest that passage of the non-polar **BB** and **SB** through the enzyme complex (metabolon) that leads to the HCH ring is rapid and is terminated by glycosylation to the much more polar glycosides, which are compartmentalised and have a greater lifetime in planta relative to the aglycones.

The observed dimeric products are believed to have arisen from an inter-molecular Diels–Alder reaction of glycosylated orthoquinols such as (**6**). Such reactions are well documented to be exquisitely regio- and stereospecific to yield totally endo-selective products in both natural and synthetic scenarios [14,15]. In nature, the [4+2] dimerization reaction of the orthoquinols is believed to be spontaneous, rather than enzyme-catalysed. We believe this is also the case for miyabeacin [3] and the new analogues reported here. The absolute configuration of these new dimeric compounds has not been directly determined here due to insufficient isolated material. However, the absolute configuration can be deduced from the proven 9(*S*) absolute configuration of the HCH group in salicortin (**1**) [12]. This stereo-centre is carried through into the dienones, such as (**6**), (**13**), and (**14**), that partake in the *endo*-specific cyclodimerisations. Natural glucosides are all D-enantiomers and the β-anomeric configuration of the glucose groups follows from the NMR coupling data. Furthermore, as we have shown [3] for miyabeacin (**3**), hydrolysis of these compounds is complex, leading ester to cleavage, decarboxylation, and also breakdown of the core via retro Diels–Alder reactions. This negates any attempt to directly determine the absolute stereochemistry of the aglycones after hydrolysis. The heritability observed in the breeding study strongly suggests genetic control over the buildup of pools of precursor dienone glycosides. The observation of products from cross-over [4+2] reactions between these (acyl)glycosylated dienones of both pathways confirms that these reactive dienones have a significant lifetime in planta. However, it remains unclear whether the inter-molecular Diels–Alder chemistry occurs in the living plant or is realised upon tissue processing and extraction of this mixture of dienone-glycosides into solvents. Further genetic analysis and careful study of engineered plants will be necessary before we can decide whether there is any involvement of a specific binding protein in accelerating the dimerisation. Also not resolved is the exact biosynthetic relationship between the dienones (salicortenone (**6**), arbusculoidenone (**13**), and their corresponding aglycones) and the saturated ketones (salicortin (**1**), arbusculoidin/isoarbusculoidin (**4**/**5**), and their aglycones). In our previous paper [3], we deduced that the genetics could not determine whether **6** (or aglycone) was a precursor that is normally reduced to **1** (or aglycone), or whether **1** (or aglycone) is hydroxylated and dehydrated to form **6** (or aglycone). These scenarios remain the same with the added complexity of the parallel benzyl pathways (**13**) and (**4**/**5**). However, what is clear is that with the judicious choice of breeding partners, new substrates can be introduced to the *Salix dasyclados* (or *S. miyabeana*)-derived ‘dienone accumulation’ trait, and, as a consequence, the subsequent spontaneous intermolecular Diels–Alder reactions can be used to produce further dimeric analogues, not only to gain insight into the biosynthetic pathway, but also to generate a panel of new analogues for further structure–activity assessment of the anti-cancer metabolite miyabeacin.

## 4. Materials and Methods

### 4.1. General Experimental Procedures

General procedures for plant tissue collection, metabolite extraction, HPLC, 600 MHz NMR spectroscopy, and UHPLC-MS have been reported previously [9]. Plant material was collected from the National Willow Collection (NWC) and from existing experimental breeding progeny growing in field plots at Rothamsted Research. Samples were harvested into liquid nitrogen and stored at −80 °C before freeze-drying. Analysis was carried out on freeze-dried, milled tissue. Tissues for metabolite screening were harvested at dormancy (January/February) or senescence (November), as described in Table 1.

### 4.2. NMR Screen of NWC Accessions and Quantification of Salicortin (***1***), Arbusculoidin (***4***), and Isoarbusculoidin (***5***)

^1^H-NMR profiling of aqueous methanol extracts was conducted at 600 MHz according to previously described methods [9]. The levels of target metabolites were quantified directly from the ^1^H-NMR data relative to an internal standard (d_4_-TSP, 0.01% *w*/*v*). Characteristic regions for each metabolite were used for the quantitation as follows: salicortin (**1**) (6.2988–6.2466, 1H/5.785–5.735, 1H); arbusculoidin (**4**) (6.6536–6.6074, 1H); and isoarbusculoidin (**5**) (5.5070–5.4727, 1H).

### 4.3. UHPLC-MS Screen for Arbusculoidin (***4***) and Miyabeacin (***3***) in Willow Breeding Progeny

Levels of arbusculoidin (**4**) and miyabeacin (**3**) were compared via UHPLC-MS in negative ionisation mode following extraction of tissue from willow breeding progeny using aqueous methanol solvent. Sample preparation and data collection were conducted as previously described [9]. Raw data files were aligned and processed using Compound Discoverer software v.3.3 (Thermo Fisher Scientific, Waltham, MA, USA) with the untargeted metabolomics workflow. Peak detection settings: mass tolerance: 10 ppm; S/N threshold: 3; min peak intensity: 500,000; values for arbusculoidin (**4**) and miyabeacin (**3**) appearing at 24.32 and 25.07 min, respectively, were extracted from the processed data table and tabulated in MS Excel.

### 4.4. Isolation and Characterisation of Arbusculoidin, Isoarbusculoidin, and Novel Dimers

For compound isolation, freeze-dried willow stem powder (240 mg) was extracted at 50 °C (10 min) in H_2_O/CH_3_OH (80:20 *v*/*v*, 4 mL). The sample was centrifuged (10 min) and the supernatant was transferred to a new tube and heated at 90 °C (2 min) to ensure that any residual hydrolytic enzyme activity was deactivated [16,17]. After cooling and centrifugation, the supernatant was moved to a glass vial for purification by HPLC. Compound isolation was carried out using an HPLC system (Dionex UltiMate 3000, Thermo Fisher Scientific) equipped with an Ascentis C-18 column (5 μm, 5 × 250 mm i.d., Supelco, Worthing, UK) maintained at 35 °C. Chromatographic separation was performed by using a constant flow rate of 1 mL/min of the mobile phases water (A) and acetonitrile (B), both containing 0.1% formic acid. The binary gradient was: 0–2 min, isocratic of 5% B; 2–10 min, linear from 5 to 22% B; 10–15 min, isocratic of 22% B; 15–25 min, linear from 22 to 30% B; 25–40.1 min, linear from 30 to 38.7, followed by 3 min wash (100% B) and 7 min re-equilibration (5% B). Peaks were detected using a diode-array UV across wavelengths of 210–360 nm and were automatically collected according to time into glass tubes. Fifteen injections (100 µL each) were performed, equivalent fractions from repeated runs were combined, and the solvent was evaporated using a Speedvac concentrator (Genevac, Suffolk, UK). UHPLC–MS was recorded for each isolated compound, in addition to 1D and 2D NMR experiments, using standard Bruker parameter sets: ^1^H-^1^H COSY (pulse sequence *cosyprqf*, ns 32), ^1^H-^1^H TOCSY (pulse sequence *mlevphpr*, ns 16), ^1^H-^1^H NOESY (pulse sequence *noesyphpr*, ns 48), ^1^H-^13^C HSQC (pulse sequence *hsqcetgpsi2*, ns 192), and ^1^H-^13^C HMBC (pulse sequence *hmbcgpndqf*, ns 256). 

### 4.5. Spectroscopic Data

See Table 2 and Table 3 for NMR data of arbusculoidin (**4**), 2′-acetylarbusculoidin (**8**), and new dimers (**9**–**12**). Compounds (**8**–**12**) had UV_max_ at 220, 254, and 270 nm.

Isoarbusculoidin (**5**): ^1^H NMR (D_2_O:CD_3_OD = 4:1) δ 7.42 (5H, m, H-2-6), 6.21 (1H, dtd, *J* = 0.9, 3.3, 9.7 Hz, H-11), 5.70 (1H, dt, *J* = 1.9, 9.7 Hz, H-10), 5.49 (1H, td, *J* = 0.7, 3.4 Hz, H-13), 5.33 (1H, d, *J* = 12.5 Hz, H-7′), 5.21 d (1H, d, *J* = 12.5 Hz, H-7′), 4.88 (1H, d, *J* = 7.8 Hz, H1′), 3.88 (1H, dd, *J* = 2.3, 12.4 Hz, H-6′), 3.65 (1H, dd, *J =* 6.2, 12.4 Hz, H-6′), 3.52 (1H, dd, *J* = 9.0, 9.5 Hz, H-4′), 3.48 (1H, ddd, *J* = 2.3, 6.2, 9.6 Hz, H-5′), 3.37–3.30 (2H, mH-2′/3′), 2.92 (2H, dd, *J* = 3.3, 5.4 Hz, H-12).

## Figures and Tables

**Table 1:** Concentrations of salicortin (**1**), arbusculoidin (**4**) and isoarbusculoidin (**5**) in stem tissue from 35 selected *Salix* species of the National Willow Collection held at Rothamsted Research, UK. Values quantified (mg/g d.w.) by ^1^H-NMR following extraction in D_2_O:CD_3_OD (4:1). n.d. = not detected.

NWC Code	Species	Clonal/ Hybrid Name	1	5	4
Dormancy					
1060	*S. gmelinii*	-	80.59 ± 1.39	7.72 ± 0.36	n.d.
1222	*S. pentandra*	E-1-1603	68.72 ± 13.43	n.d.	14.35 ± 0.91
1270	*S. triandra*	1978	n.d.	20.05 ± 0.95	n.d.
1198	*S. fragilis*	259-5	78.83 ± 3.63	n.d.	8.15 ± 0.88
1239	*S. saposhnikovii*	19197	16.55 ± 1.43	33.25 ± 3.05	12.73 ± 2.12
1102	615 *S. schwerinii* ‘K3 Hilliers’ × ‘Jorr’	Endeavour	47.32 ± 2.15	n.d.	13.11 ± 0.19
1123	615 *S. schwerinii* ‘K3 Hilliers’ × ‘Bjorn’	Discovery	38.43 ± 4.02	n.d.	16.97 ± 0.60
1124	SW900812 × ‘Quest’	Resolution	n.d.	n.d.	35.35 ± 1.76
1156	*S. acutifolia*.	Vitellina	74.45 ± 8.89	n.d.	8.72 ± 0.25
1283	*S. triandra* × *dasyclados*	884	58.41 ± 1.08	n.d.	13.77 ± 0.55
191	*S. alba*	Kew	51.31 ± 2.77	n.d.	12.71 ± 0.56
208	*S. alba* var ‘Typica’	Padova	59.05 ± 2.44	n.d.	13.11 ± 1.66
210	*S. alba* var. coerulea	Wantage Hall	59.98 ± 2.93	n.d.	16.01 ± 1.40
215	*S. alba* var. coerulea	Oath	68.23 ± 1.57	n.d.	11.18 ± 0.85
216	*S. alba* var. coerulea	Forres	54.91 ± 1.54	n.d.	14.69 ± 1.84
237	*S. alba* var. *sericea*	-	29.44 ± 0.99	n.d.	8.85 ± 1.06
395	*S. fragilis*	Oeil Noir	110.42 ± 17.29	n.d.	9.79 ± 0.28
577	*S. dasyclados*	77056 IEA Trial	70.77 ± 4.44	3.83 ± 0.30	n.d.
615	*S. schwerinii*	K3 Hilliers	n.d.	n.d.	16.87 ± 1.52
635	*S. viminalis* × *schwerinii*	Olof	40.12 ± 6.66	n.d.	9.97 ± 0.41
648	*S. viminalis*	Vigorous	37.03 ± 3.79	n.d.	9.63 ± 1.40
741	*S. turanica*	-	16.79 ± 0.02	n.d.	8.79 ± 0.87
890	*S. tenuijulis*	-	111.27 ± 51.49	n.d.	24.04 ± 1.39
1165	*S. arbusculoides*	20397	n.d.	24.15 ± 1.89	18.82 ± 1.02
638	1128 ‘Pavainen’ × ‘Bjorn’	Quest	24.78 ± 2.19	n.d.	18.33 ± 1.11
628	*S. schwerinii* ‘L79069’ × ‘Orm’	Tora	11.22 ± 0.45	n.d.	15.94 ± 0.89
636	‘Astrid’ × ‘Bjorn’	Asgerd	16.91 ± 0.77	n.d.	6.23 ± 0.16
1092	*S. viminalis*	L810203	13.32 ± 0.61	n.d.	3.05 ± 0.08
1095	SW930887 × ‘Bjorn’	Sherwood	20.44 ± 1.02	n.d.	21.40 ± 1.66
640	SW911066 × ‘Jorr’	Inger	18.25 ± 0.87	n.d.	6.16 ± 0.18
662	*S. viminalis*	Longifolia	15.67 ± 0.34	n.d.	14.51 ± 0.94
1132	*(S. viminalis × viminalis) × (S. viminalis × schwerinii)*	SW930812	11.02 ± 0.11	n.d.	23.80 ± 2.53
Senescence					
1102	615 *S. schwerinii* ‘K3 Hilliers’ × ‘Jorr’	Endeavour	23.64 ± 1.73	n.d.	9.93 ± 0.69
577	*S. dasyclados*	77056	26.07 ± 1.21	4.15 ± 0.72	n.d.
614	*S. schwerinii*	CE 78-22	n.d.	n.d.	31.62 ± 1.40
617	*S. schwerinii*	Henrik	n.d.	n.d.	33.23 ± 1.25
619	*S. dasyclados × schwerinii*	Lapin	18.05 ± 1.77	n.d.	15.32 ± 0.38
621	*S. schwerinii*	Siberian 077	10.01 ± 0.26	n.d.	27.58 ± 0.46
632	‘Tora’ × ‘Orm’	Torhild	13.32 ± 0.55	n.d.	11.08 ± 0.08

**Table 2:** NMR data for arbusculoidin **4** and its 2′-acetyl analogue **8** (D_2_O:CD_3_OD, 4:1 containing d_4_-TSP (0.01% *w*/*v*)) δ in ppm relative to d_4_-TSP at 0.00.

Position	Arbusculoidin (4)			2′-Acetyl Arbusculoidin (8)		
	δ ^13^C	δ ^1^H	*J*_H-H_ (Hz); Multiplicity	δ ^13^C	δ ^1^H	*J*_H-H_ (Hz); Multiplicity
9	83.0	-	-	83.3	-	-
10	124.9	5.77	1.7, 10.0; *dt*	125.2	5.74	1.7, 10.0; *dt*
11	141.8	6.63	4.2, 10.0; *dt*	142.0	6.60	4.2, 10.0; *dt*
12	26.5	2.62 2.54	*m* *m*	27.2	2.59	*m*
13	39.6	2.75	3.0, 6.7; *td*	39.7	2.75	2.8, 6.5; *td*
14	212.6	-	-	211.9	-	-
8	172.4	-	-	172.2	-	-
1	137.7	-	-	137.8	-	-
2	131.7	7.43	*m*	131.8	7.43	*m*
3	131.7	7.43	*m*	131.8	7.43	*m*
4	131.2	7.35	1.7, 7.7; *dd*	131.4	7.36	1.6, 7.7; *dd*
5	131.7	7.43	*m*	131.8	7.43	*m*
6	131.7	7.43	*m*	131.8	7.43	*m*
7	71.4	5.25 5.23	12.2; *d* 12.2; *d*	71.4	5.24 5.20	12.2; *d* 12.2; *d*
1′	100.7	4.71	7.5; *d*	98.6	4.90	8.0; *d*
2′	75.8	3.41	7.5, 8.5; *dd*	76.4	4.77	8.0, 9.5; *dd*
3′	78.4	3.45	*m*	76.8	3.62	9.5; *d*
4′	72.4	3.36	*m*	72.2	3.44	9.0, 9.5; *dd*
5′	79.1	3.29	*m*	79.2	3.32	*m*
6′	63.3	3.77 3.62	2.1, 12.5; *dd* 5.3, 12.5; *dd*	63.3	3.78 3.65	2.1, 12.5; *dd* 5.4, 12.5; *dd*
CH_3_**C**O-	*-*	-	-	176.0	-	-
**CH_3_**CO-	*-*	-	-	23.3	2.03	*s*

**Table 3:** NMR data for dimers **9**, **10**, **11**, and **12** (D_2_O:CD_3_OD, 4:1 containing d_4_-TSP (0.01% *w*/*v*)) δ in ppm relative to d_4_-TSP at 0.00.

	9		10		11		12	
Position	δ ^1^H (*J*_H-H_ (Hz))	δ ^13^C	δ ^1^H (*J*_H-H_ (Hz))	δ ^13^C	δ ^1^H (*J*_H-H_ (Hz))	δ ^13^C	δ ^1^H (*J*_H-H_ (Hz))	δ ^13^C
1	-	158.0	-	158.2	-	157.8	-	158.2
2	-	127.1	-	127	-	127.3	-	127.1
3	7.34 *dd* (1.5, 7.6)	133.2	7.33 *dd* (1.6, 7.6)	133.7	7.37 *m*	132.0	7.34 *dd* (1.6, 7.6)	133.7
4	7.12 *td* (0.8, 7.6)	125.9	7.11 *td* (0.9, 7.6)	125.8	7.14 *td* (0.7. 7.5)	126.1	7.12 *td* (0.9, 7.5)	125.9
5	7.39 *m*	132.2	7.41 *m*	132.5	7.40 *m*	133.6	7.40 *m*	131.9
6	7.20 *dd* (0.7, 8.4)	118.0	7.20 *d* (0.7, 8.2)	118	7.21 *d* (8.3)	118.2	7.21 *dd* (0.9, 8.0)	118.1
7	5.37 *d* (12.1) 5.15 *d* (12.1)	66.7	5.41 *d* (11.9) 5.18 *d* (11.9)	67	5.34 *d* (12.2) 5.22 *d* (12.2)	66.2	5.41 *d* (12.0) 5.21 *d* (12.0)	67.0
8	-	170.4	-	173.8		170.7	-	173.8
9	-	88.5	-	82.2		86.2	-	82.2
10	3.87 *dd* (1.6, 8.0)	40.3	3.69 *dd* (1.5, 8.2)	40.2	3.74 *m*	40.4	3.57 *m*	40.0
11	3.46 *m*	43.5	3.59 *m*	43.5	3.41 *m*	43.3	3.52 *m*	43.7
12	6.51 *dd* (4.2, 10.2)	150.9	6.61 *dd* (4.1, 10.2)	152.3	6.44 *dd* (3.8. 10.2)	149.8	6.63 *dd* (4.0, 10.2)	152.3
13	6.03 *dd* (1.5, 10.2)	132.0	6.08 *dd* (1.7, 10.2)	131.2	6.05 *dd* (1.3. 10.2)	132.6	6.08 *dd* (1.7, 10.2)	131.4
14	-	197.5	-	198.6	-	198.0	-	198.7
15	3.70 *m*	45.0	3.90 *dt* (1.7, 6.6)	43.8	3.56 *m*	44.4	3.78 *m*	43.8
16	6.18 *ddd* (0.8, 6.6, 7.8)	136.0	6.23 *ddd* (1.0, 6.6, 7.8)	136.4	6.00 *t* (7.9)	135.3	6.21 *ddd* (1.2, 7.2, 8.0)	136.0
17	5.91 *ddd* (1.4, 6.2, 7.8)	133.2	5.92 *ddd* (1.3, 6.2, 7.8)	132.3	5.93 *ddd* (1.4, 7.0, 8.1)	132.9	5.94 *ddd* (1.3, 6.5, 7.9)	132.6
18	3.41 *m*	53.9	3.47 *m*	53.9	3.41 *m*	53.8	3.45 *ddd* (1.3, 2.5, 6.3)	54.2
19	-	210.0	-	207.2	-	210.2	-	206.3
20	-	80.0	-	85.5	-	79.9	-	84.4
21	-	173.6		169.7	-	173.4	-	169.9
Glc-1	5.08 *d* (7.4)	103.2	5.06 *d* (7.8)	103.3	5.07 *d* (7.4)	103.2	5.08 *d* (7.7)	103.3
Glc-2	3.56 *m*	76.0	3.54 *dd* (7.8, 9.4)	76.6	3.56 *m*	76.0	3.55 *m*	76.1
Glc-3	3.59 *m*	78.8	3.59 *m*	78.8	3.58 *m*	78.8	3.60 *m*	79.0
Glc-4	3.50 *d* (8.9)	72.5	3.48–3.55 *m*	73.5	3.48 *dd* (8.7. 9.6)	72.3	3.51 *m*	72.6
Glc-5	3.59 *m*	78.9	3.59 *m*	78.8	3.58 *m*	78.8	3.60 *m*	79.0
Glc-6	3.92 *dd* (2.2, 12.5) 3.73 *dd* (5.9, 12.5)	63.7	3.94 *dd* (2.2, 12.5) 3.77 *dd* (5.5, 12.5)	63.8	3.91 *dd* (2.2. 12.3) 3.73 *dd* (5.5. 12.2)	63.4	3.93 *dd* (2.1, 12.4) 3.77 *dd* (5.6, 12.3)	63.7
1′	-	137.4	-	137.5		137.4	-	137.7
2′	7.43 *m*	132.1	7.42 *m*	131.7	7.43 *m*	131.8	7.43 *m*	131.8
3′	7.43 *m*	132.1	7.42 *m*	132.5	7.43 *m*	131.8	7.43 *m*	131.8
4′	7.39 *m*	132.3	7.39 *m*	132.2	7.37 *m*	132.0	7.40 *m*	131.9
5′	7.43 *m*	132.1	7.42 *m*	132.5	7.43 *m*	131.8	7.43 *m*	131.8
6′	7.43 *m*	132.1	7.42 *m*	131.7	7.43 *m*	131.8	7.43 *m*	131.8
7′	5.30 *d* (11.9) 5.22 *d* (11.9)	72.1	5.27 *d* (12.1) 5.15 *d* (12.1)	71.7	5.29 *d* (12.0) 5.24 *d* (12.1)	71.9	5.23 *s*	71.7
Glc-1′	4.35 *d* (7.6)	103.2	4.71 *d* (7.5)	102.2	4.80 (obscured)	100.3	4.94 *d* (8.0)	100.1
Glc-2′	3.42 *m*	76.1	3.31 *m*	75.8	4.76 *dd* (8.0. 9.4)	76.2	4.66 *dd* (8.0, 9.7)	76.5
Glc-3′	3.59 *m*	78.8	3.59 *m*	78.3	3.56 *m*	76.0	3.49 *dd* (9.7, 8.9)	76.3
Glc-4′	3.37 *m*	71.8	3.50 *m*	72.5	3.44 *m*	72.1	3.32 *m*	72.2
Glc-5′	2.94 *ddd* (2.1, 4.6, 9.4)	79.0	3.25 *m*	79.1	3.07 *ddd* (2.2. 5.2. 9.6)	79.0	3.22 *ddd* (2.1, 5.6, 9.8)	79.6
Glc-6′	3.71 *dd* (2.1, 12.3) 3.58 *m*	63.3	3.78 *dd* (2.0, 12.5) 3.49 *dd* (5.0, 12.5)	63.8	3.70 *m* 3.59 *m*	63.0	3.74 *dd* (2.1, 12.3) 3.50 *m*	63.5
**CH_3_**CO-	-	-	-	-	2.12 *s*	23.1	2.10 *s*	23.3
CH_3_**C**O-	-	-	-	-	-	175.8	-	175.8

## Data Availability

The original contributions presented in the study are included in the article/supplementary material, and further inquiries can be directed to the corresponding authors.

## Supplementary Materials

The following supporting information can be downloaded at: https://www.mdpi.com/article/10.3390/plants13121609/s1, Figure S1**:** LCMS data (negative ion mode) following extraction with 80:20 water:methanol; Figure S2: LC-MS data (negative ion mode) for Miyabeacin (**3**) and acetyl miyabeacin (**7**); Figure S3: LC-MS data (negative ion mode) for diacetyl miyabeacin; Figure S4: LC-MS data (negative ion mode) for hybrid dimers (**9**) and (**10**) formed via Diels–Alder reaction between salicortenone (**6**) and arbusculoidenone (**13**); Figure S5: LC-MS data (negative ion mode) for acetylated hybrid dimers (**11**) and (**12**) formed via Diels–Alder reaction between salicortenone (**6**) and 2′-acetylarbusculoidenone (**14**); Figure S6: LC-MS data (negative ion mode) for acetylated hybrid dimers formed via Diels–Alder reaction between 2′-acetylsalicortenone and arbusculoidenone (**13**); Figure S7: LC-MS data (negative ion mode) for putative diacetylated hybrid dimers formed via Diels–Alder reaction between 2′-acetylsalicortenone and 2′-acetylarbusculoidenone; Figure S8: LC-MS data (negative ion mode) for dimeric analogue generated from a Diels–Alder reaction of two arbusculoidenone monomers; Figure S9: LC-MS data (negative ion mode) for putative monoacetylated arbusculoidenone-based dimers; Figure S10: LCMS data (negative ion mode) following extraction with 80:20 water:methanol. Quantitation (peak area) of characteristic ([M − H]^−^/[M + FA − H]^−^) ions of monomer metabolites and dimeric products in RR10143 and its parental and grandparental germplasm; Table S1: Quantified peak areas from willow breeding progeny (Trial “RR/CS/722”) of arbusculoidin and miyabeacin.
