# Supplementary material for: Breeding Novel Chemistry in Willow: New Hetero Diels–Alder Cyclodimers from Arbusculoidin and Salicortin Suggest Parallel Biosynthetic Pathways
Source: Plants (Basel). 2024 Jun 11;13(12):1609. doi: 10.3390/plants13121609 (PMC11207313; doi:10.3390/plants13121609)
Supplement: Supplementary file 1 [file plants-13-01609-s001.zip › plants-3003026-supplementary.pdf]

Supplementary Information

Figure S1: LCMS data (negative ion mode) following extraction with 80:20 water:methanol. A: Total ion chromatogram of NWC615; B: Total ion chromatogram of NWC1239; C: Extracted ion chromatogram for m/z 453 for NWC615; D: Extracted ion chromatogram for m/z 453 for NWC615; E: Mass spectrum of m/z 453 at 24.32 min. F: MSMS spectrum of m/z 407 at 24.32 min; G: MSMS spectrum of m/z 453 at 21.88 min

Figure S2: LC-MS data (negative ion mode) for miyabeacin **(3)** and acetylmiyabeacin **(7).** A: extracted ion chromatogram for m/z 843 (miyabeacin); B: mass spectra indicating parent ion and formate adduct; C: monomer ion formed via in source fragmentation; D: extracted ion chromatogram for m/z 885 (acetyl miyabeacin); E: mass spectra indicating parent ion and formate adduct; F: monomer ions formed via in source fragmentation

Figure S3: LC-MS data (negative ion mode) for diacetyl miyabeacin**.** A: extracted ion chromatogram for m/z 927 (diacetyl miyabeacin); B: mass spectra indicating parent ion and formate adduct; C: monomer ion formed via in source fragmentation;

Figure S4: LC-MS data (negative ion mode) for hybrid dimers (**9**) and (**10**) formed via Diels Alder reaction between salicortenone (**6**) and arbusculoidenone (**13**). A: extracted ion chromatogram for m/z 827; B: mass spectra indicating parent ion and formate adduct; C: monomer ions formed via in source fragmentation.

Figure S5: LC-MS data (negative ion mode) for acetylated hybrid dimers (**11**) and (**12**) formed via Diels Alder reaction between salicortenone (**6**) and 2′-acetylarbusculoidenone (**14**). A: extracted ion chromatogram for m/z 869; B: mass spectra indicating parent ions and formate adduct; C: monomer ions formed via in source fragmentation.

Figure S6: LC-MS data (negative ion mode) for acetylated hybrid dimers formed via Diels Alder reaction between acetyl salicortenone and arbusculoidenone (**13**). A: extracted ion chromatogram for m/z 869; B: mass spectra indicating parent ions and formate adduct; C: monomer ions formed via in source fragmentation.

Figure S7: LC-MS data (negative ion mode) for putative diacetylated hybrid dimers formed via Diels Alder reaction between 2′-acetylsalicortenone and 2′-acetylarbusculoidenone. A: extracted ion chromatogram for m/z 911; B: mass spectra indicating parent ion and formate adduct; C: monomer ions formed via in source fragmentation.

Figure S8: LC-MS data (negative ion mode) for dimeric analogue generated from a Diels Alder reaction of two arbusculoidenone monomers**.** A: extracted ion chromatogram for m/z 811; B: mass spectra indicating parent ion; C: monomer ion formed via in source fragmentation;

Figure S9: LC-MS data (negative ion mode) for putative monoacetylated arbusculoidenone based dimers. A: extracted ion chromatogram for m/z 953; B: mass spectra indicating parent ion; C: monomer ions formed via in source fragmentation.

Figure S10: LCMS data (negative ion mode) following extraction with 80:20 water:methanol. Quantitation (peak area) of characteristic ($[M\text{-}H]^-$/$[M\text{+}FA\text{-}H]^-$) ions of monomer metabolites and dimeric products in RR10143 and its parental and grand-parental germplasm.

Table S1. Quantified peak areas from willow breeding progeny (Trial “RR/CS/722”) of arbusculoidin and miyabeacin

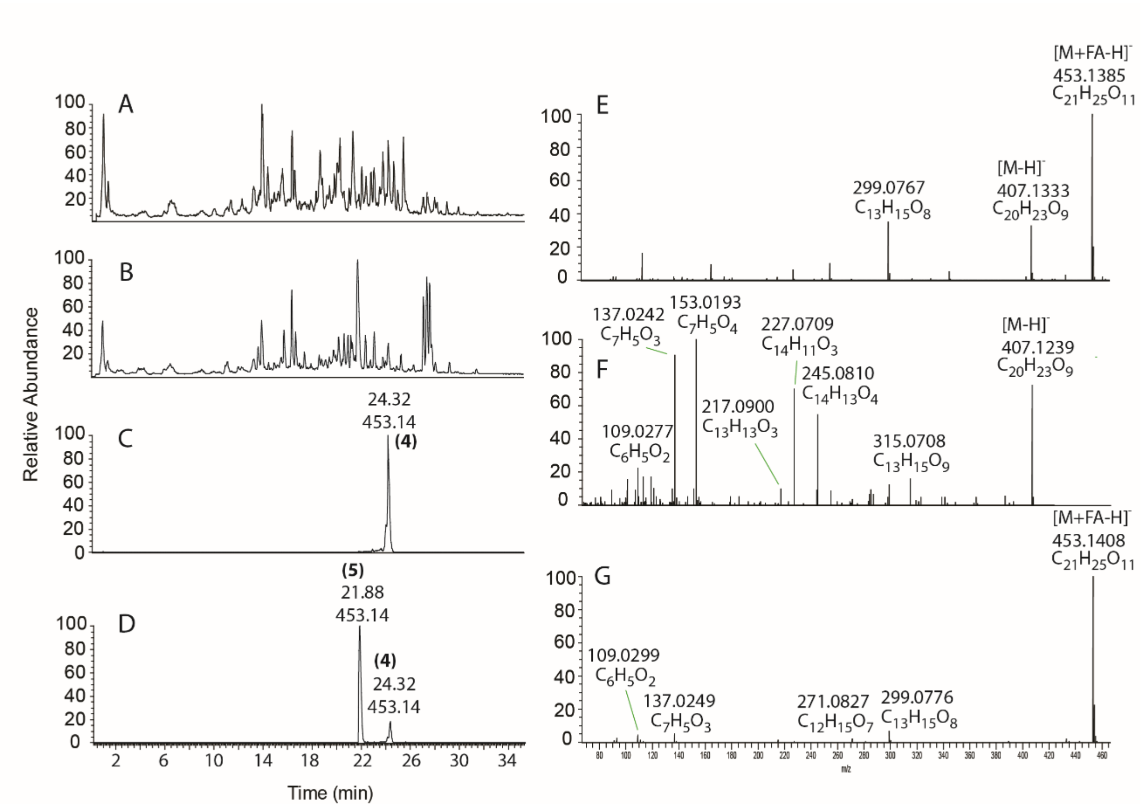

Figure S1: LCMS data (negative ion mode) following extraction with 80:20 water:methanol. A: Total ion chromatogram of NWC615; B: Total ion chromatogram of NWC1239; C: Extracted ion chromatogram for m/z 453 for NWC615; D: Extracted ion chromatogram for m/z 453 for NWC615; E: Mass spectrum of m/z 453 at 24.32 min. F: MSMS spectrum of m/z 407 at 24.32 min; G: MSMS spectrum of m/z 453 at 21.88 min

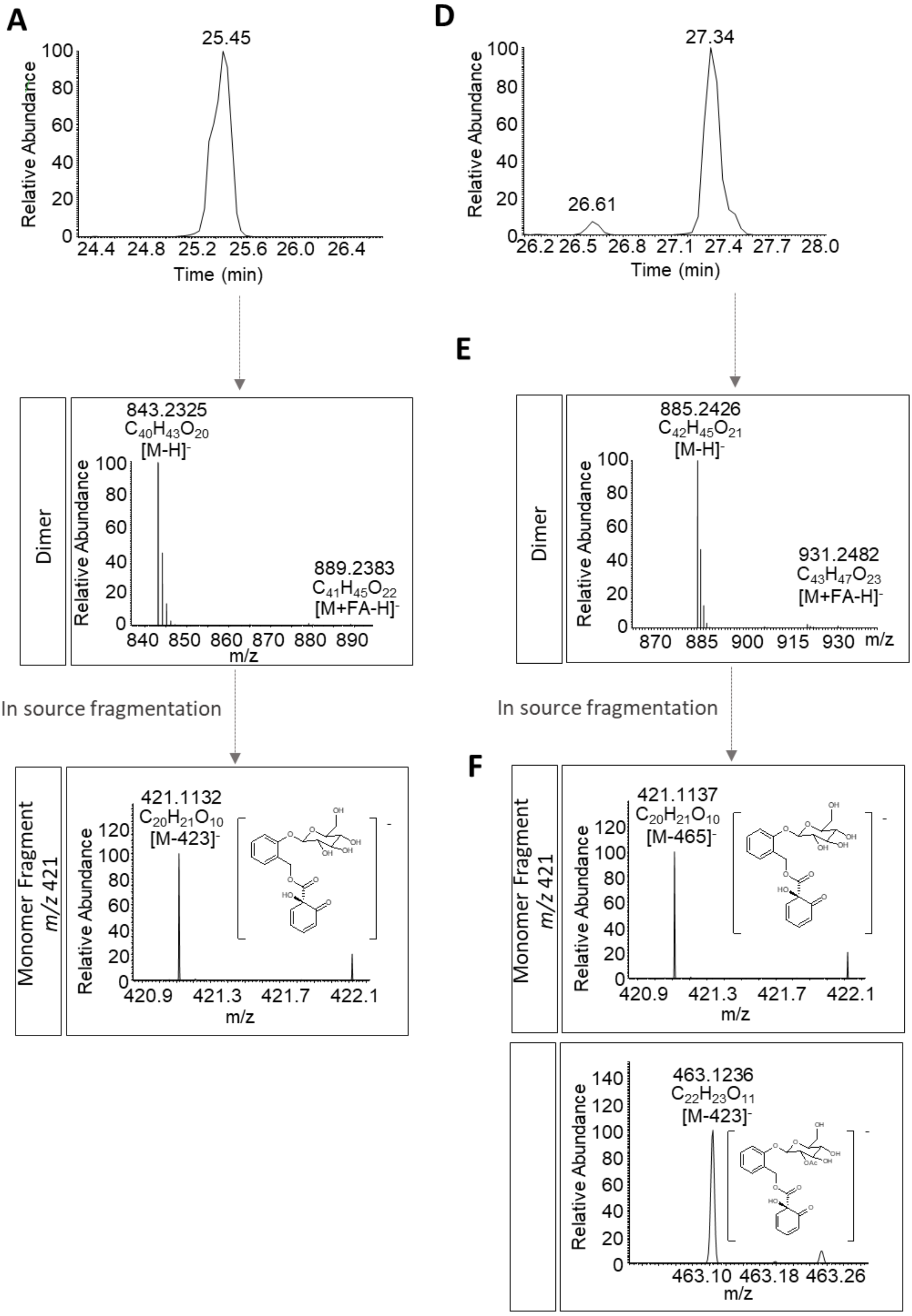

Figure S2: LC-MS data (negative ion mode) for miyabeacin **(3)** and acetylmiyabeacin **(7).** A: extracted ion chromatogram for m/z 843 (miyabeacin); B: mass spectra indicating parent ion and formate adduct; C: monomer ion formed via in source fragmentation; D: extracted ion chromatogram for m/z 885 (acetyl miyabeacin); E: mass spectra indicating parent ion and formate adduct; F: monomer ions formed via in source fragmentation

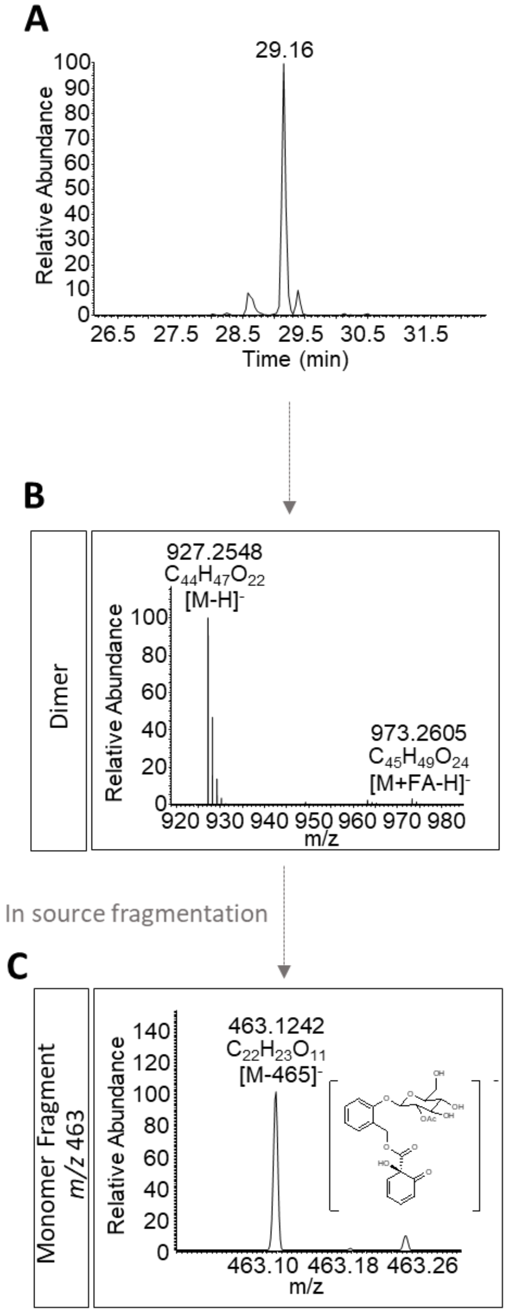

Figure S3: LC-MS data (negative ion mode) for diacetyl miyabeacin**.** A: extracted ion chromatogram for m/z 927 (diacetyl miyabeacin); B: mass spectra indicating parent ion and formate adduct; C: monomer ion formed via in source fragmentation;

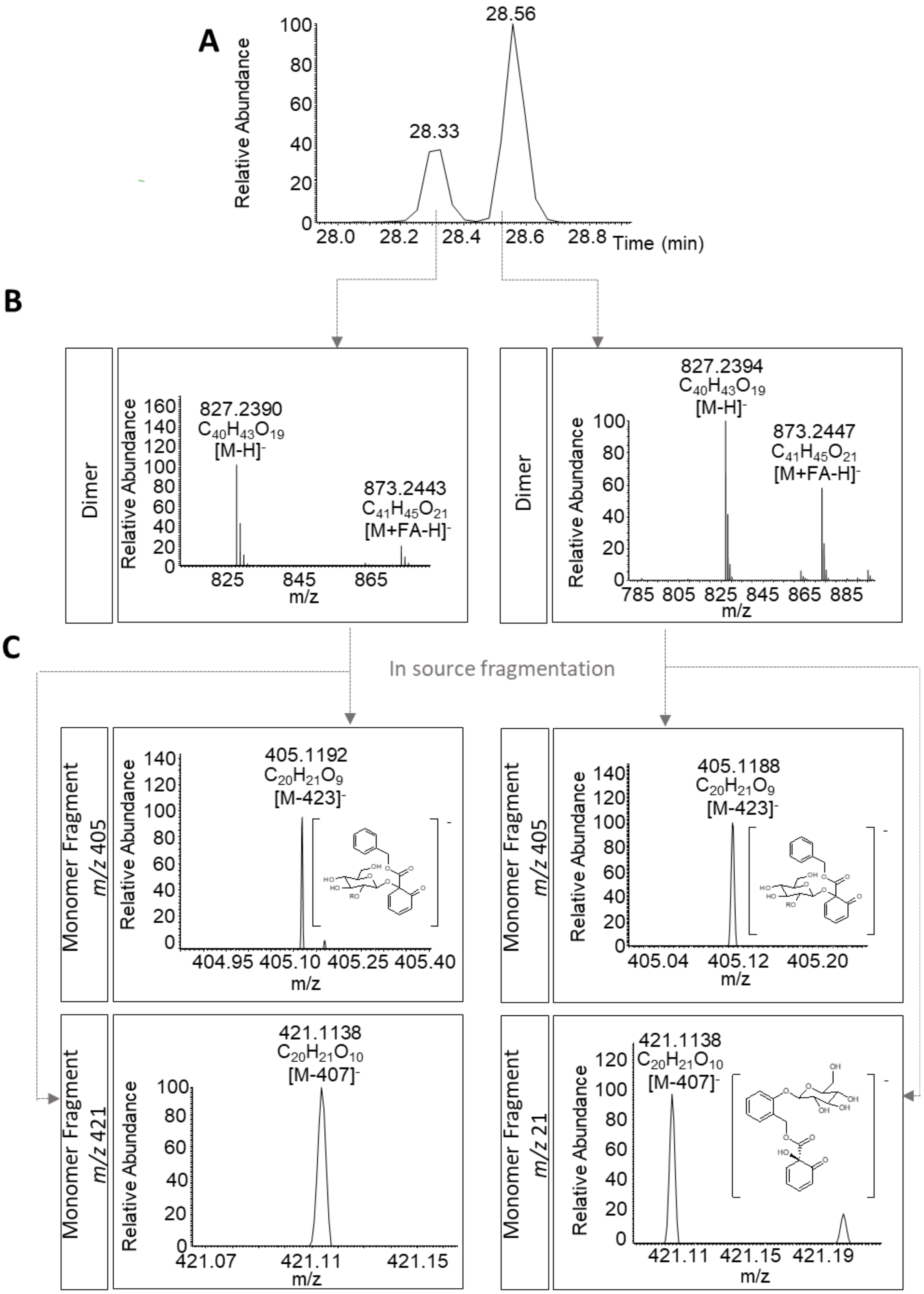

Figure S4: LC-MS data (negative ion mode) for hybrid dimers (**9**) and (**10**) formed via Diels Alder reaction between salicortenone (**6**) and arbusculoidenone (**13**). A: extracted ion chromatogram for m/z 827; B: mass spectra indicating parent ion and formate adduct; C: monomer ions formed via in source fragmentation.

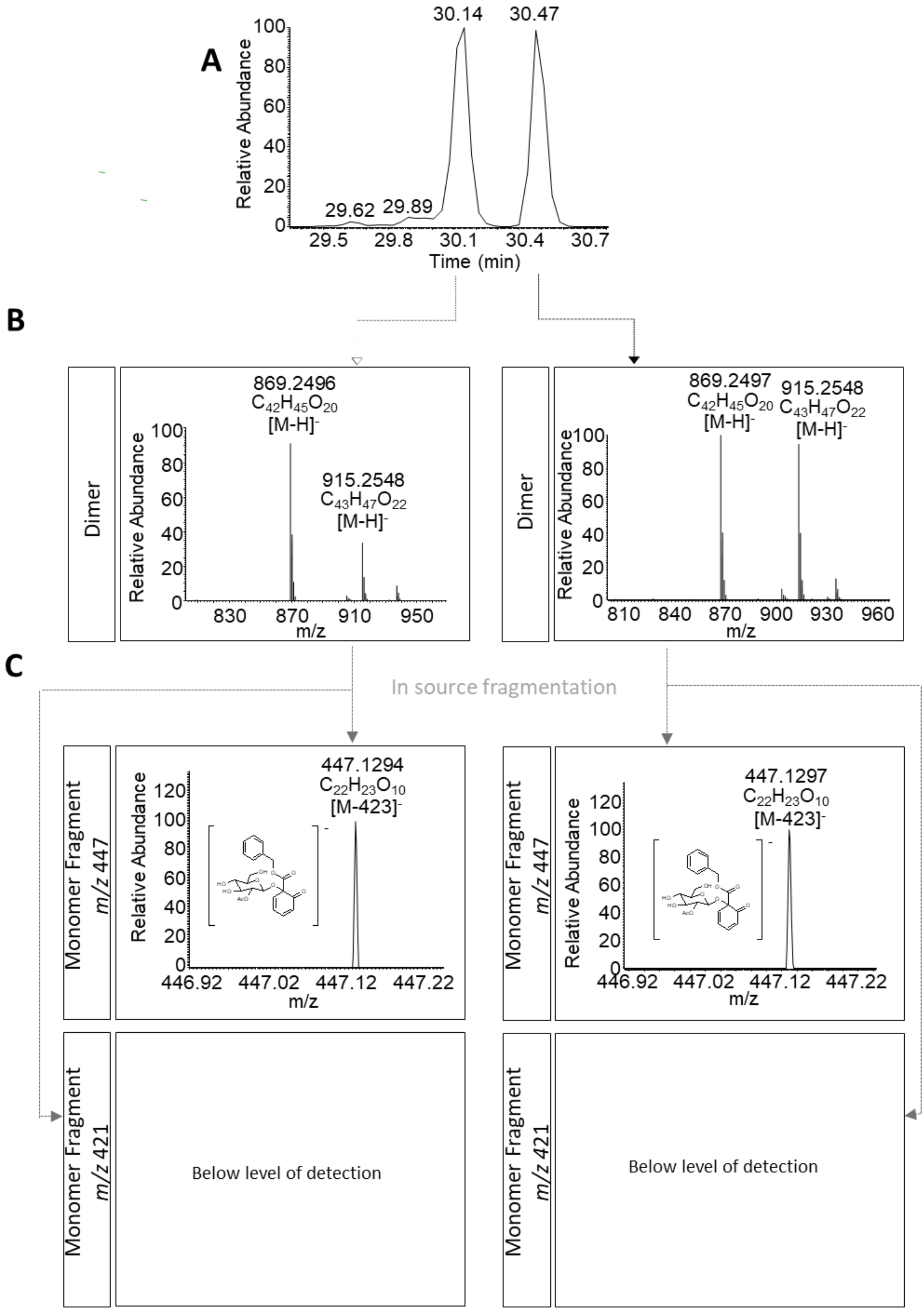

Figure S5: LC-MS data (negative ion mode) for acetylated hybrid dimers (**11**) and (**12**) formed via Diels Alder reaction between salicortenone (**6**) and 2′-acetyl arbusculoidenone (**14**). A: extracted ion chromatogram for m/z 869; B: mass spectra indicating parent ions and formate adduct; C: monomer ions formed via in source fragmentation.

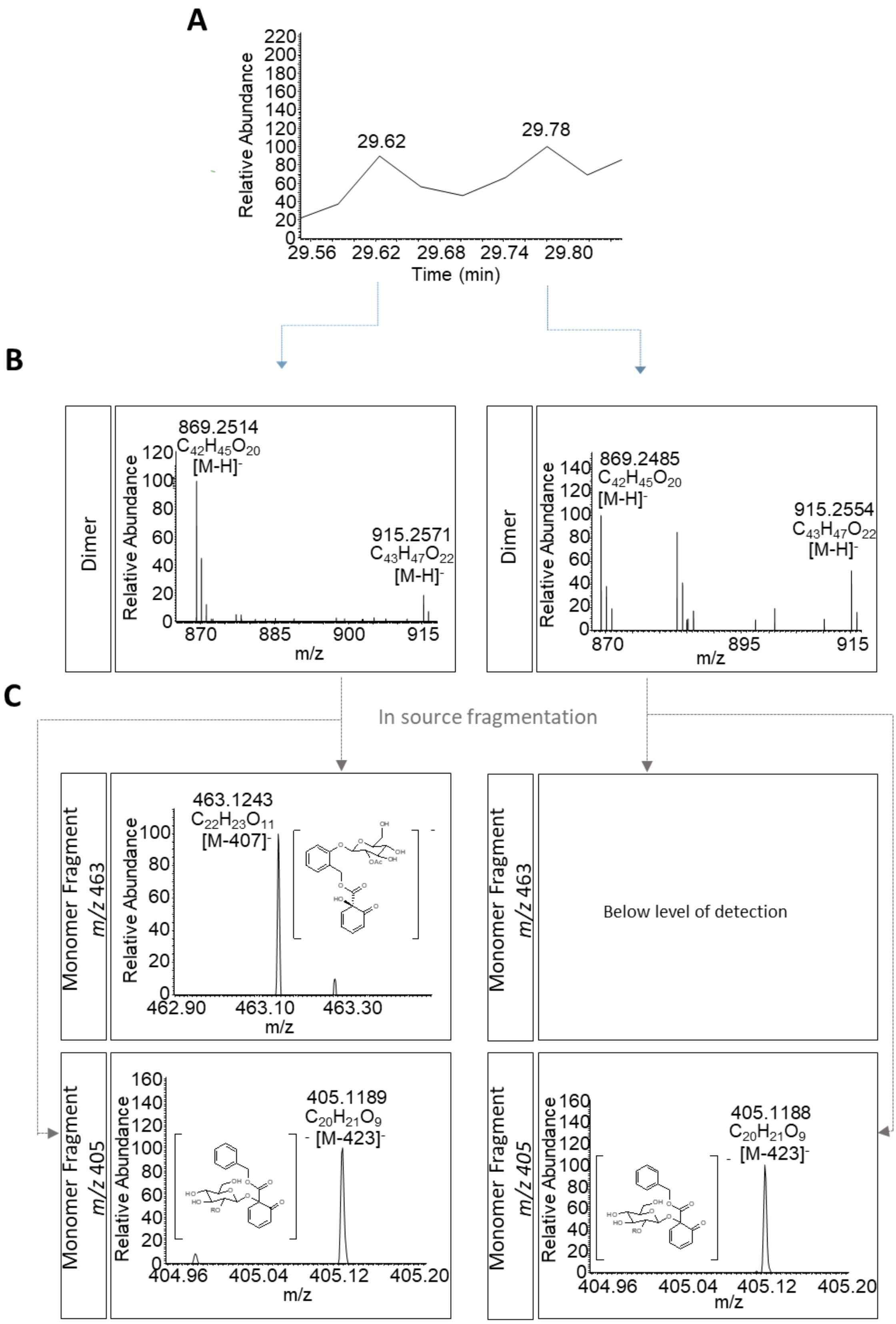

Figure S6: LC-MS data (negative ion mode) for acetylated hybrid dimers formed via Diels Alder reaction between 2′-acetylsalicortenone and arbusculoidenone (**13**). A: extracted ion chromatogram for m/z 869; B: mass spectra indicating parent ions and formate adduct; C: monomer ions formed via in source fragmentation.

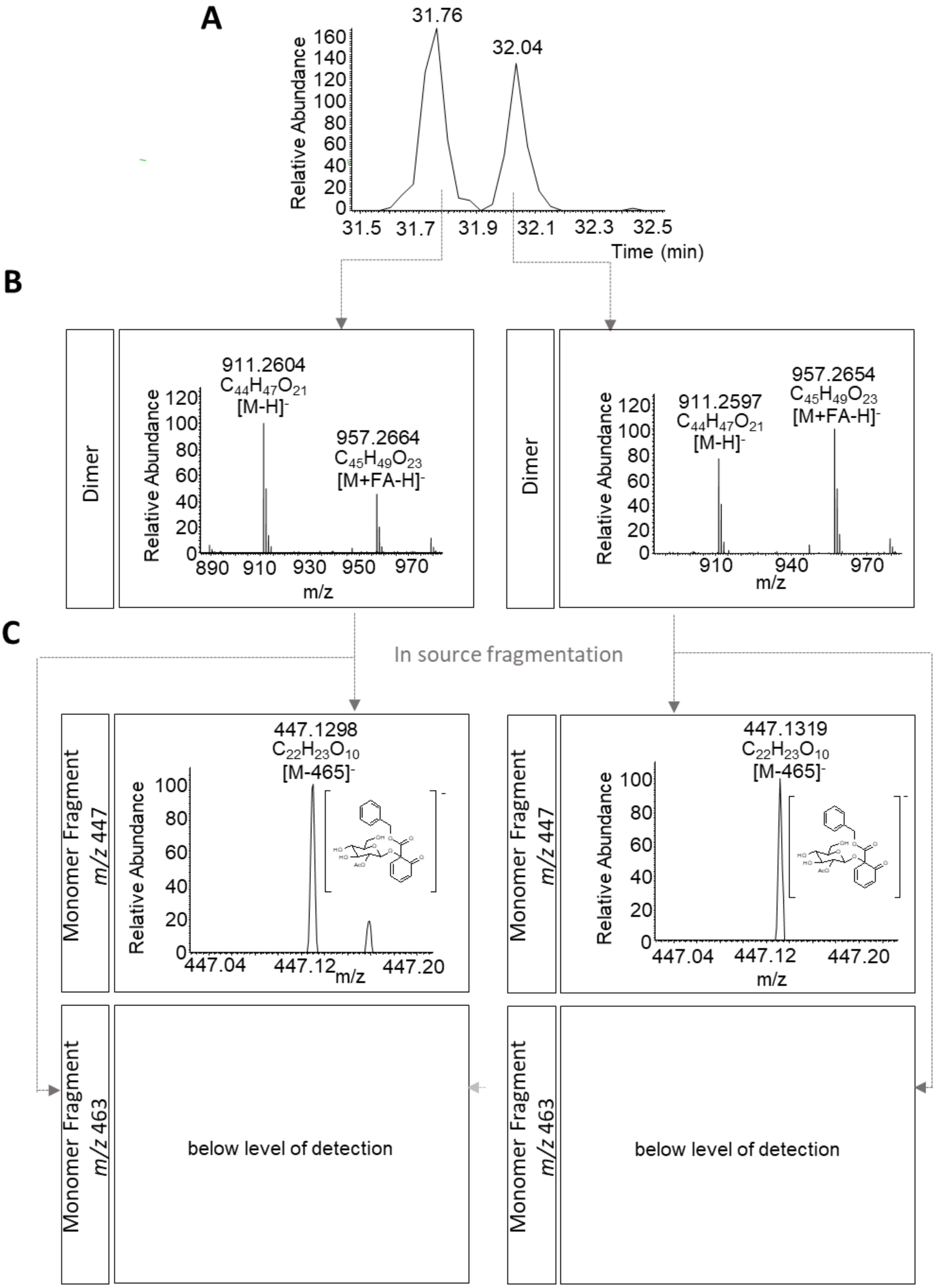

Figure S7: LC-MS data (negative ion mode) for putative diacetylated hybrid dimers formed via Diels Alder reaction between acetyl salicortenone and 2’-acetylarbusculoidenone. A: extracted ion chromatogram for m/z 911; B: mass spectra indicating parent ion and formate adduct; C: monomer ions formed via in source fragmentation.

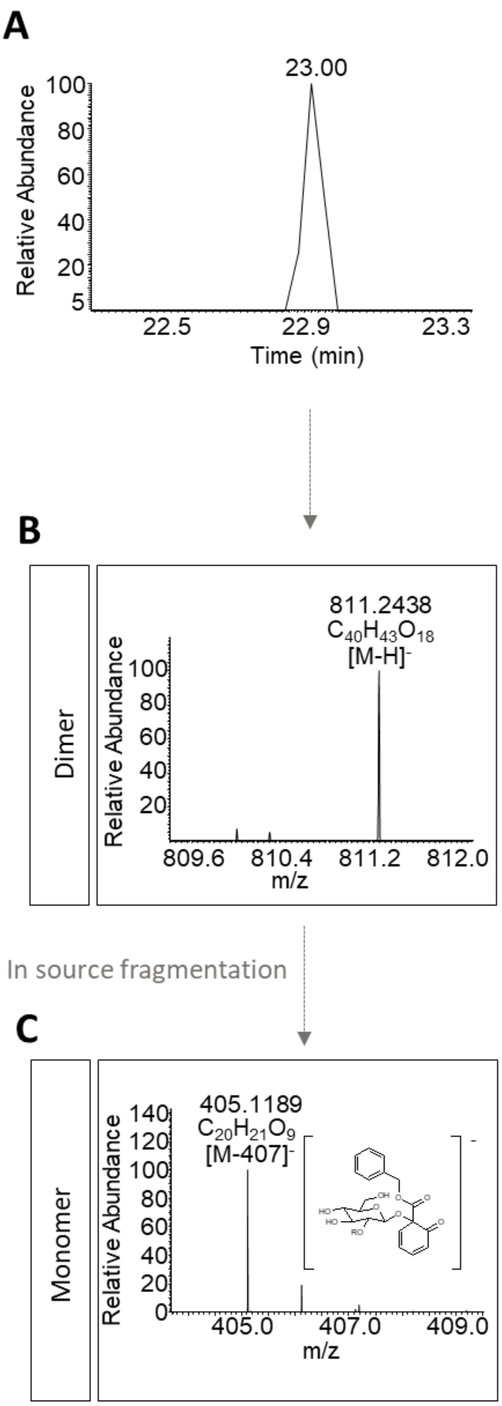

Figure S8: LC-MS data (negative ion mode) for dimeric analogue generated from a Diels Alder reaction of two arbusculoidenone monomers**.** A: extracted ion chromatogram for m/z 811; B: mass spectra indicating parent ion; C: monomer ion formed via in source fragmentation;

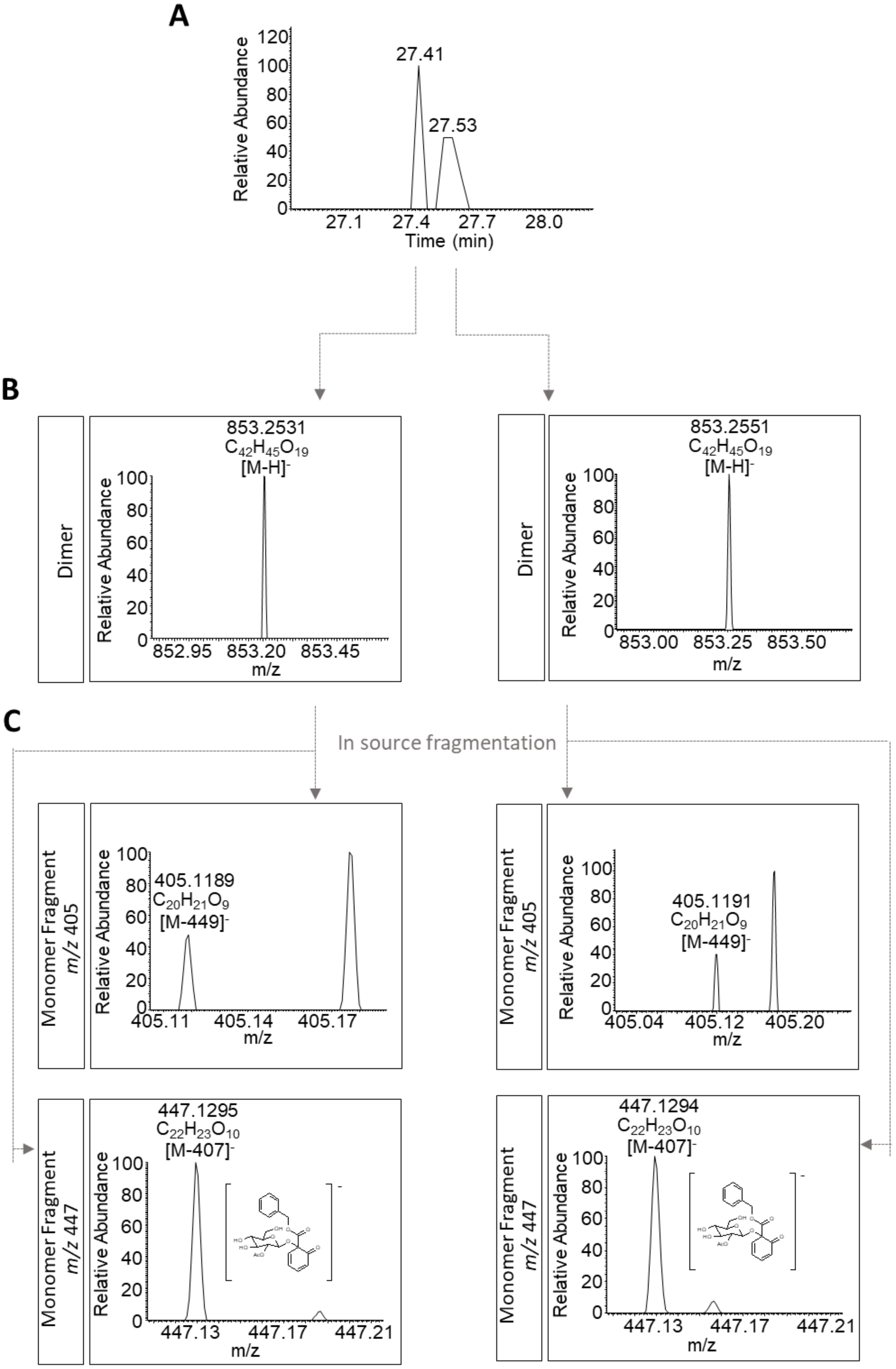

Figure S9: LC-MS data (negative ion mode) for putative monoacetylated arbusculoidenone based dimers. A: extracted ion chromatogram for m/z 953; B: mass spectra indicating parent ion; C: monomer ions formed via in source fragmentation.

	NWC607	NWC577	NWC619	RR10143
**Salicortin (1)**	596532878	579112032	449561155	507659977
**Acetyl salicortin (2)**	1741597233	10399029	152306942	178998469
**Arbusculoidin (4)**	0	2908072	56686652	14107340
**Acetylarbusculoidin (8)**	0	0	0	14020453
**Isoarbusculoidin (5)**	0	59961228	0	65575091
**Miyabeacin (5)**	0	163884390	484754	144580603
**Acetyl miyabeacin (7)**	0	8721287	0	38495557
**[9]**	0	0	0	2052458
**[10]**	0	0	0	6468344
**[11]**	0	0	0	2451921
**[12]**	0	0	0	3040352

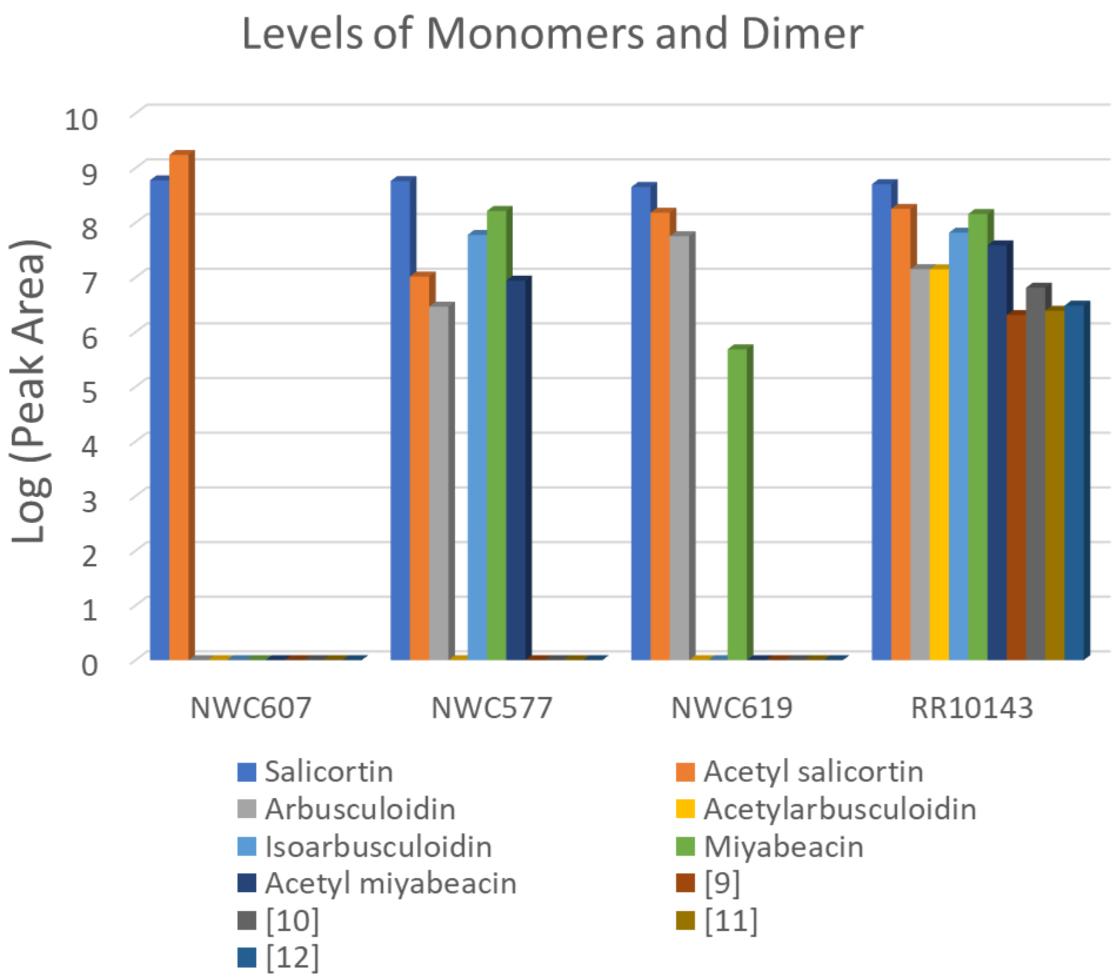

Figure S10: LCMS data (negative ion mode) following extraction with 80:20 water:methanol. Quantitation (peak area) of characteristic ($[M-H]^-$/$[M+FA-H]^-$) ions of monomer metabolites and dimeric products in RR10143 and its parental and grand-parental germplasm.

Table S1. Quantified peak areas from willow breeding progeny (Trial “RR/CS/722”) of arbusculoidin and miyabeacin

Breeders Code	Arbusculoidin	Miyabeacin	Parents
**RR10038**	71025695	337116	RR08083 (607 *S. rehderiana* × 619 (*S. dasyclados* × *schwerinii*) ‘Lapin’) × 446 *S. aegyptiaca*
**RR10036**	65349327	268765	RR08083 (607 *S. rehderiana* × 619 (*S. dasyclados* × *schwerinii*) ‘Lapin’) × 446 *S. aegyptiaca*
**RR10236**	64017621	44331012	RR08083 (607 *S. rehderiana* × 619 (*S. dasyclados* × *schwerinii*) ‘Lapin’) × 885 *S. miyabeana* ‘Shrubby’
**RR10034**	63299059	12712400	RR08083 (607 *S. rehderiana* × 619 (*S. dasyclados* × *schwerinii*) ‘Lapin’) × 446 *S. aegyptiaca*
**RR10051**	62045638	363295	RR08083 (607 *S. rehderiana* × 619 (*S. dasyclados* × *schwerinii*) ‘Lapin’) × 446 *S. aegyptiaca*
**RR10043**	44740299	0	RR08083 (607 *S. rehderiana* × 619 (*S. dasyclados* × *schwerinii*) ‘Lapin’) × 446 *S. aegyptiaca*
**RR10045**	42636321	1783143	RR08083 (607 *S. rehderiana* × 619 (*S. dasyclados* × *schwerinii*) ‘Lapin’) × 446 *S. aegyptiaca*
**RR10042**	38020810	1346939	RR08083 (607 *S. rehderiana* × 619 (*S. dasyclados* × *schwerinii*) ‘Lapin’) × 446 *S. aegyptiaca*
**RR10140**	29957230	116816076	RR08083 (607 *S. rehderiana* × 619 (*S. dasyclados* × *schwerinii*) ‘Lapin’) × 577 *S. dasyclados* '77056'
**RR10044**	26973878	419150	RR08083 (607 *S. rehderiana* × 619 (*S. dasyclados* × *schwerinii*) ‘Lapin’) × 446 *S. aegyptiaca*
**RR10143**	24543438	126169615	RR08083 (607 *S. rehderiana* × 619 (*S. dasyclados* × *schwerinii*) ‘Lapin’) × 577 *S. dasyclados* '77056'
**RR10321**	24167355	82296945	RR08054 ('Tordis’ × *S. triandra* 'Semperflorens') × *S. miyabeana* ‘Shrubby’
**RR10091**	21269482	92432921	‘Tordis’ × 941 *S. miyabeana* ‘Purpurescens’
**RR10223**	20877315	122624375	RR04248 (‘Discovery’ × ‘Quest’) × 577 *S. dasyclados* ‘77056’
**RR10316**	19060527	63491218	RR06088 (‘Tora’ × 1011 *S. viminalis*) × 575 *S. dasyclados* ‘Loden’
**RR07162**	18964892	91289915	‘Torhild’ (‘Tora’ x ‘Orm’) x 941 *S. miyabeana* ‘Purpurescens’
**RR10340**	18899499	130849297	RR05326 (‘Resolution’ × 609 *S. rossica*) × 941 *S. miyabeana* ‘Purpurescens’
**RR10341**	18778656	114241543	RR05326 (‘Resolution’ × 609 *S. rossica*) × 941 *S. miyabeana* ‘Purpurescens’
**RR10317**	18062637	88773472	RR06088 (‘Tora’ × 1011 *S. viminalis*) × 575 *S. dasyclados* ‘Loden’
**RR10339**	16568301	100876068	RR05326 (‘Resolution’ × 609 *S. rossica*) × 941 *S. miyabeana* ‘Purpurescens’
**RR10346**	16214017	112389176	RR05326 (‘Resolution’ × 609 *S. rossica*) × 941 *S. miyabeana* ‘Purpurescens’
**RR10342**	15963461	96574153	RR05326 (‘Resolution’ × 609 *S. rossica*) × 941 *S. miyabeana* ‘Purpurescens’
**RR10145**	15448875	67416583	RR08083 (607 *S. rehderiana* × 619 (*S. dasyclados* × *S. schwerinii*) ‘Lapin’)) × 577 *S. dasyclados* '77056'
**RR10348**	15260749	95789712	RR05326 (‘Resolution’ × 609 S. *rossica*) × 941 *S. miyabeana* ‘Purpurescens’
**RR10337**	15011366	136355036	RR05326 (‘Resolution’ × 609 S. *rossica*) × 941 *S. miyabeana* ‘Purpurescens’
**RR10222**	14964301	84238603	RR04248 (‘Discovery’ × ‘Quest’) × 577 *S. dasyclados* ‘77056’
**RR10335**	13402671	62525780	RR05326 (‘Resolution’ ×609 *S. rossica*) × 941 *S. miyabeana* ‘Purpurescens’

**RR10349**	13216743	106303111	RR05326 (‘Resolution’ × 609 *S. rossica*) × 941 *S. miyabeana* ‘Purpurescens’
**RR10336**	12619137	93005908	RR05326 (‘Resolution’ × 609 *S. rossica*) × 941 *S. miyabeana* ‘Purpurescens’
**RR10221**	12565834	96634281	RR04248 (‘Discovery’ × ‘Quest’) × 577 *S. dasyclados* ‘77056’
**RR10345**	12273815	114280512	RR05326 (‘Resolution’ × 609 *S. rossica*) × 941 *S. miyabeana* ‘Purpurescens’
**RR10084**	11017730	117423448	‘Tordis’ × 941 *S. miyabeana* ‘Purpurescens’
**RR10350**	10845314	89901231	RR05326 (‘Resolution’ × 609 *S. rossica*) × 941 *S. miyabeana* ‘Purpurescens’
**RR10338**	10797570	52374669	RR05326 (‘Resolution’ × 609 *S. rossica*) × 941 *S. miyabeana* ‘Purpurescens’
**RR10347**	9928865	159084212	RR05326 (‘Resolution’ × 609 *S. rossica*) × 941 *S. miyabeana* ‘Purpurescens’
**RR07110**	9811003	35708621	‘Torhild’ (‘Tora’ x ‘Orm’) x 575 S. *dasyclados* ‘Loden’
**RR10343**	8189424	113136690	RR05326 (‘Resolution’ × 609 *S. rossica*) × 941 *S. miyabeana* ‘Purpurescens’
**RR10344**	6847618	114279172	RR05326 (‘Resolution’ × 609 *S. rossica*) × 941 *S. miyabeana* ‘Purpurescens’
**RR10098**	3533243	54669842	RR07187 (944 *S. glaucophyloides* × 577 *S. dasyclados* ‘77056’) × 577 *S. dasyclados* ‘77056’
**RR07155**	3027996	33106083	459 *S. caprea* ‘Smithiana’ x 577 *S. dasyclados* ‘77056’
**RR10213**	2922080	48249034	RR06131 (971 *S. eriocephela* × 1028 *S. hookeriana*) × 575 *S. dasyclados* ‘Loden’
**RR10214**	2744085	37392168	RR06131 (971 *S. eriocephela* × 1028 *S. hookeriana*) × 575 *S. dasyclados* ‘Loden’
**RR07196**	2294722	97160614	944 *S. glaucophyloides* x 575 *S. dasyclados* ‘Loden’
**RR10147**	2265008	59879230	RR07187 (944 *S.* glaucophyloides × 577 *S. dasyclados* ‘77056’) × RR07188 (944 *S. glaucophyloides* × 577 *S. dasyclados* ‘77056’)
**RR10151**	2028694	3300435	RR07187 (944 *S. glaucophyloides* × 577 *S. dasyclados* ‘77056’) × RR07188 (944 *S. glaucophyloides* × 577 *S. dasyclados* ‘77056’)
**RR10096**	1171020	75676167	RR07187 (944 *S.* glaucophyloides × 577 *S. dasyclados* ‘77056’) × 577 *S. dasyclados* ‘77056’
**RR10095**	900548	92370705	RR07187 (944 *S. glaucophyloides* × 577 *S. dasyclados* ‘77056’) × 577 *S.* dasyclados ‘77056’
**RR10087**	735240	113791408	‘Tordis’ × 941 *S. miyabeana* ‘Purpurescens’
**RR10215**	696524	19599203	RR06131 (971 *S. eriocephela* × 1028 *S. hookeriana*) × 575 *S. dasyclados* ‘Loden’
**RR10150**	656727	30448866	RR07187 (944 *S. glaucophyloides* × 577 ‘77056’) × RR07188 (944 *S. glaucophyloides* × 577 *S. dasyclados* ‘77056’)
**RR10191**	649708	88567848	RR06141 (954 *S. rigida* × 575 *S. dasyclados* ‘Loden’) × 577 *S. dasyclados* ‘77056’
**RR10194**	294376	76235905	RR06141 (954 *S. rigida* × 575 *S. dasyclados* ‘Loden’) × 577 *S. dasyclados* ‘77056’
**RR10193**	252762	76136721	RR06141 (954 *S. rigida* × 575 *S. dasyclados* ‘Loden’) × 577 *S. dasyclados* ‘77056’
**RR10035**	0	836271	RR08083 (607 *S. rehderiana* × 619 (*S. dasyclados* × *schwerinii*) 'Lapin') × 446 *S. aegyptiaca*

**RR10037**	0	251651	RR08083 (607 *S. rehderiana* × 619 (*S. dasyclados* × *schwerinii*) 'Lapin') × 446 *S. aegyptiaca*
**RR10039**	0	0	RR08083 (607 *S. rehderiana* × 619 (*S. dasyclados* × *schwerinii*) 'Lapin') × 446 *S. aegyptiaca*
**RR10040**	0	0	RR08083 (607 *S. rehderiana* × 619 (*S. dasyclados* × *schwerinii*) 'Lapin') × 446 *S. aegyptiaca*
**RR10041**	0	443187	RR08083 (607 *S. rehderiana* × 619 (*S. dasyclados* × *schwerinii*) 'Lapin') × 446 *S. aegyptiaca*
**RR10046**	0	1408503	RR08083 (607 *S. rehderiana* × 619 (*S. dasyclados* × *schwerinii*) 'Lapin') × 446 *S. aegyptiaca*
**RR10047**	0	345576	RR08083 (607 *S. rehderiana* × 619 (*S. dasyclados* × *schwerinii*) 'Lapin') × 446 *S. aegyptiaca*
**RR10048**	0	2651225	RR08083 (607 *S. rehderiana* × 619 (*S. dasyclados* × *schwerinii*) 'Lapin') × 446 *S. aegyptiaca*
**RR10049**	0	1088332	RR08083 (607 *S. rehderiana* × 619 (*S. dasyclados* × *schwerinii*) 'Lapin') × 446 *S. aegyptiaca*
**RR10050**	0	725570	RR08083 (607 *S. rehderiana* × 619 (*S. dasyclados* × *schwerinii*) 'Lapin') × 446 *S. aegyptiaca*
**RR10118**	0	67347432	446 *S. aegyptiaca* × 941 *S. miyabeana* ‘Purpurescens’
**RR10120**	0	50924669	446 *S. aegyptiaca* × 941 *S. miyabeana* ‘Purpurescens’
**RR10123**	0	55135910	446 *S. aegyptiaca* × 941 *S. miyabeana* ‘Purpurescens’
**RR10238**	0	27159265	RR08083 (607 *S. rehderiana* × 619 (*S. dasyclados* × *schwerinii*) 'Lapin') × 885 *S. miyabeana* ‘Shrubby’
**RR10239**	0	7447749	RR08083 (607 *S. rehderiana* × 619 (*S. dasyclados* × *schwerinii*) 'Lapin') × 885 *S. miyabeana* ‘Shrubby’
**RR10323**	0	31195851	RR08054 (‘Tordis’ 99 *S. triandra* 'Semperflorens') × 885 *S. miyabeana* ‘Shrubby’
**RR10326**	0	53862222	RR08054 (‘Tordis’ × 99 *S. triandra* 'Semperflorens') × 885 *S. miyabeana* ‘Shrubby’

Notes:

-Identification of individual accessions from the National Willow Collection have been made based on a number of characteristic phenotypic assessments, verified by multiple experts. To the best of our knowledge, they were correct at the time of the generation of breeding progeny. A full genotypic assessment of the collection is currently underway and will be published in due course. Accession numbers (NWCXXX) will remain unchanged regardless of any re-classification.

RR10118, RR10120 & RR10123 - 446 *S. aegyptiaca* is a hermaphroditic willow species. In the breeding programme 446 S. *aegyptiaca* has been used as a male and female parent. In this case emasculation was performed to allow the cross to made in this direction.

-RR04248 is the breeders code for ‘Roth Chiltern’
